# Application of Krylov–Bogoliubov–Mitropolski method to asymmetric gyrostatic 3D motion in multi-fields

**DOI:** 10.1038/s41598-025-27177-5

**Published:** 2025-11-27

**Authors:** T. S. Amer, A. H. Elneklawy, H. F. El-Kafly

**Affiliations:** 1 Department of Mathematics, Faculty of Science, Tanta University, Tanta, 31527 Egypt; 2 Department of Mathematics, Faculty of Science, Kafrelsheikh University, Kafr El-Sheikh, 33516 Egypt; 3 Tanta Higher Institute of Engineering and Technology, Tanta, Egypt

**Keywords:** Rigid body, Euler–Possion equations, Perturbation methods, Euler angels, Nonlinear dynamics, Krylov–Bogoliubov–Mitropolski method, Multi fields, Applied mathematics, Computational astrophysics

## Abstract

The 3D rotary movement of an asymmetric rigid body (RB) in space around a fixed point is investigated. A gyrostatic torque (GT), a magnetic field (MF), and a Newtonian force field (NFF) all have an impact on the RB’s ability to rotate. Around its minor axis of inertia, the body is thought to begin moving rapidly. The Krylov–Bogoliubov–Mitropolski (KBM) method is used to solve the governing equations of motion (EOMs) analytically once they are created using Euler–Poisson equations. Additionally, Euler angles approximate analytical solutions (AS) are examined. A graphical simulated viewpoint of the obtained results and the equations of Euler angles, which show how the body is orientated at each given moment, are used to debate the interpretation of the motion. Maintaining control over the body’s rotation and position during motion can be achieved by researching the effects of different values of the acting forces and toques, such as the GT, MF, and NFF. To illustrate how the stability of the RB is affected, phase graphs of various solutions have been created. Periodicity behavior is demonstrated by the closed-phase curves’ symmetry around any of their axes. Together with the beneficial impacts of these forces and torques, the movement behavior of the RB is evaluated and simulated. The results obtained are widely relevant to gyroscopic equipment, especially those that incorporate inertial systems like aircraft and satellites. Technologies that guarantee the motion stability of applications are also included.

**Supplementary Information:**

The online version contains supplementary material available at 10.1038/s41598-025-27177-5.

## Introduction

Many scholars have for years concentrated their attention on RB’s problem-solving, despite the fact that it is one of the most challenging problems in theoretical mechanics. Understanding the motions of numerous objects in our daily lives requires an understanding of the rotatory movement of RBs. Researchers can better predict the long-term viability and stability of RBs in a variety of scenarios by examining their rotation. In the domains of engineering and design, where accurate rotational motion is essential to the functionality and safety of machinery and structures, this understanding is especially important. Furthermore, understanding rotational motion enhances the general efficiency and maximizes the effectiveness of mechanical systems. In the end, having a solid grasp of rotational motion enables us to develop and improve technology for a variety of uses.

The earliest mention of this issue dates back to 1758 when Euler established its basic specifications and looked at the RB’s free motion, that is, motion without the body’s torque. In 1788 and 1889, respectively, the tops of Lagrange and Kovalevskaya were investigated in a gravity environment. It is significant to notice that Euler–Poisson’s equations, which govern the motion, consist of six nonlinear differential equations (DEs) that allow one to obtain three first integrals^1^. After establishing a further independent fourth first integral under the criteria of the preceding three ones, it was possible to prove that these equations are integrable. This problem has numerous integrable scenarios that may be found in^2,3^. In^4^, for the matrix of rotation of a free asymmetric RB with an angular velocity vector resting on separatrices, the author evaluates Poisson equations and finds their perfect solution in fundamental functions. In^5^, for the situation of a charged RB, an overall solution to the EOMs is found. Finding the overall solution is simplified to the issue of a 1D cubic pseudo-oscillator for the case of a symmetrical charged substance. In^6^, a novel approach to solving the EOMs of a system with three rotors rotating around the primary axes of the RB is put forth. The GT satisfies Poisson equation when the multiplier is constant, and the area integral is used to determine how it changes. For the gyro and the RB’s motions, novel solutions of these EOMs are found when the mass distribution is spherical. In weakly elliptical orbits^7^, the study finds steady periodic orientation whose period coincides with the period of the orbit close to hyperbolic revolution. The numerical simulations of the nonlinear orientation dynamics of the RB are used to construct and evaluate approximated analytical solutions for these periodical motions. According to stability examination, inner and outer compound resonances may cause unsteadiness, whereas non-resonant circumstances produce steady periodic orientation motions. In^8^, the study introduced an approximate representation of the dynamic solution of the RB that is nearly dynamically spherical and contains a viscoelastic element. By employing an asymptotic approach, the authors were able to derive good outcomes and utilizing computations and reduced averaged formulas, chart the course of angle movement. It offers a unique contribution through its initial computational and asymptotic estimates, providing estimations that accurately give a minimally inaccurate description of the long-term development of motion during a limitless time range for the RB with a movable mass. In^9^, the RB’s perturbed rotational motions under the influence of a torque that varies gradually over time are examined, approaching the Lagrange scenario^9^. For dynamically symmetric rotating RBs^10^, the authors have derived an approximate solution to the system of Euler’s equations with an extra perturbation term. In^11^, the authors have approached the fourth first integral for the problem in the Euler, Lagrange, and Kovalevskaya scenarios with the influence of the GT on the motion.

In the recent era, the use of perturbation methods and Homotopy perturbation methods^12,13^ has become widespread among researchers to find the problem’s AS, which results from the challenge of acquiring the fourth integral in a generic form. One of these methods is the Poincaré method of small parameter (PMSP) which has been used in several works. Some of these approaches are mentioned in^14,15^, where the author examined the scientific developments in the field of RB dynamics over the last five decades. The exact solution for freely spinning movement of the RB with a rotor of little power is examined in^16^. Depending on the characteristics of the problem and the beginning circumstances, the author demonstrated that the carrier body’s movement is nearly equivalent to rotation about a fixed axis. In^17^, for the situation of the RB model that is subjected to a time-varying heating field, the traditional Euler–Poisson equations are reviewed. It is demonstrated that while the energy integral should be adjusted in accordance with the gradually shifting primary torques of inertia resulting from the influenced by temperature diameters of the RB, two fundamental first integrals for movement stay unchanged. In^18^, the rotary motion of an asteroid is examined. The authors discovered that while the kinetic spin of the asteroid is reduced by energy dissipation, various perturbations can disrupt the asteroid’s rotation, leading it to deviate from its current spin state. Consequently, the asteroid’s rotation should stabilize around the axis with the greatest inertia, preserving a constant angular momentum and achieving the spin state with minimal energy. In^19^, the particle’s momentum equations of motion are solved through a mathematical technique, yielding an exact solution for three nonlinear ordinary DEs corresponding to the velocity components of a charged particle. The system is then solved semi-analytically for specific cases of parametrically variable MFs. In^20^, the EOMs of a dancing top are generally solved in terms of the exponentially of the Hamiltonian field. An additional constraint that considers the supplementary plane’s reaction modifies the canonical Poisson structure, raising doubts about the integration based on Liouville.

The approach of KBM is thought to be among the crucially significant techniques to acquire the AS of RB’s DEs. In comparison to PMSP, this approach has a substantial benefit for figuring out approximations for the RB problems as; since the solutions are independent of the periodicity criteria, we don’t need to be familiar with them beforehand. Moreover, the secular terms must be eliminated to identify the unidentified functions that emerge during the solution. The convergence of the sequence of acquired solutions is considered a problem for the PMSP, but it’s not regarded as a problem for the approach of KBM. Therefore, it has been possible to acquire the RB motion’s AS via several subsequent attempts^21,22^. This approach is used in^21^ to attain a typical AS in the uniform gravitational field (UGF) and NFF for the RB motion-controlling system. These solutions have been shown to have singular points, as mentioned before. In^22^, these singularities have been accomplished (in the UGF and the presence of two components of GT) is taken into account.

In^23^, an analysis focuses on the rotating movement of a charged RB enclosing a spherical chamber that is filled with a dense liquid. That study takes into account the effects of GT, constant torques acting on the RB’s axes (CBFTs), and a torque generated by an opposing force because of the liquid’s geometry. The averaging method is employed to simplify the governing EOMs. Taylor’s method is employed to resolve the averaged equations, considering specific initial circumstances in order to attain the intended results. By combining the asymptotic approach with numerical analysis, the study is able to accurately determine the results of the problem. In^24^, by using the established correlation, the inertial torques cause the gyroscope to rotate. Their direction and shape at the spatially coordinate system determine inertial tension. There is a mechanical inaccuracy in the well-known analytical model for the rotating disc’s axis of rotation. The centrifugal inertial torque was not properly integrated, resulting in this mistake. The precise expression for the interacting rotations of the rotating disc about axes is obtained from the adjusted inertial torque^25^. examined a new AS for the rotatory movement of a charged axisymmetric spinning RB when subjected to a GT. The impact of both transverse and CBFTs, as well as the MF, are also taken into account. In^26^, a study approached three distinct cases for the solution of the same problem on the main three inertial axes for various values of the CBFTs and under the influence of the GT, and^27^ introduced a novel solution using special functions. In^28^, a symmetrical gyrostat with a center of mass moving in an elliptical orbital in a central NFF is shown to be stable under third- and fourth-order resonances. Resonant spin is a unique flat cycle of movement of the gyrostat about its center of mass, meaning that throughout two orbital cycles of its center of mass, the body completes a single revolution in unrestricted space. In^29^, a computational representation of a gyrostat system with three rotors and a rigid exterior framework is set up, taking into account exterior torque and frictional torque. For pressure evaluation, this framework is converted into a Kolmogorov-type system. When the gyrostat is subjected to an exterior torque in addition to interior, dissipative, and inertial torques, a four-wing chaotic vortex is discovered. The primary contribution of^30^ is the suggestion of a structure-preserving iteration technique to examine how the dynamic symmetry-breaking elements affect the rigid-flexible coupling systems’ dynamic behaviors. In^31^, the vehicle-bridge interaction problem is studied using the extended multi-symplectic method, a common structure-preserving technique for the infinite-dimensional non-conservative systems. In^32^, a reduced coupled nonlinear dynamic model based on continuum mechanics and the Hamiltonian variational principle is presented for the flexible hub-beam system revolving around an eccentric axis.

In^33^, the PMSP is used to derive the solution for a symmetric model of the RB. The case of irrational frequencies has been studied and the solution for the governing EOMs has been addressed. In^34^, the primary goal is to build upon earlier findings regarding the dynamic movement of a symmetric RB under the implications of perturbation and restoring torques. By utilizing an averaging method, the study derives the system of EOMs for the averaged dynamics. The approximated solution is then approached for the problem achieving, its main purpose. In^35^, a novel method is proposed for solving the problem of a nearly symmetrical RB model. The authors assumed that the body rotates with constant angular velocity along its minor axes, which simplifies Euler’s equations into decoupled linear differential equations, later solved. Additionally, they employed a well-known special function to approximate the analytical solutions. In^36,37^, the authors study the rotary motion of Goryachev–Sretensky gyrostat. By using Chetaev’s technique^36^ and establishing that one of the angular velocity components rotates having zero average value for all solutions that are not part of the region of the integral’s zero level set, every stationary solution has been located. While in^37^, the stability of each stationary solution is examined when they are located on the invariant set of the area integral’s zero level. The action of the GT is of a particular kind in the situation where the suspension points, and the center of mass coincide. The exact solutions for the problem have been approached and shown in^38,39^. It has been shown how these derived solutions are affected by the GT, and the varying torques in^38^ as the phase portraits have also been studied to observe the regularity and stability of the angular velocities. In^40–43^, Euler-Poisson’s equations that define the EOMs of how the RB rotates around a stationary point under the influence of the GT have been examined. The exact solution to the problem is provided and graphically depicted using computer codes, which allow us to analyze the motion at any given time. The effect of GT distinctive values on these solutions is also covered in^39^, while the effect of energy dissipation has been shown in^40,41^. In^42^, a structure-preserving approach is used to examine the coupling dynamic behaviors of the tethered satellite system, which is idealized as a planar flexible damping beam-spring-mass composite system. In^43^, the analytical solution for the problem is approached using the PMSP. In^44^, the authors discuss a gyrostat satellite in a small inclination, weakly elliptical near-Earth orbit. The gyrostat has an intrinsic magnetic torque, electrostatic charge, and a flywheel. It is investigated how the gyrostat attitude motion is affected by Lorentz, magnetic, and gravity-gradient torques. In^45^, control torques based on Lorentz forces are used to stabilize regular precessions of a satellite in a circular orbit. A substantially asymmetrical satellite’s rapid rotating motion with respect to the center of mass is examined in^46,47^. Gravity torques and minor pressure forces cause the satellite to move. It contains a hollow filled with a viscous fluid at low Reynolds numbers. In^48^, an axially symmetric gyrostat satellite’s rotational motion in a circular orbit while subjected to its gravitational pull is examined. Investigations are conducted on periodic movements of the satellite’s symmetry axis with respect to the orbital coordinate system.

This study seeks to look into the 3D rotatory motion of an asymmetric RB around one of its fixed points in a general case. The body is considered to rotate under the impact of NFF, MF, and GT about the RB’s main axis. It is hypothesized that around the third main axis, the beginning angular velocity of RB is substantial. The approach of KBM is accustomed to obtaining AS of the governing EOMs. Euler’s angles, which show how the body is oriented at each given instant, and the graphic representation of the obtained solution, are used to frame the discussion of the motion’s interpretation. The solutions’ phase graphs, which illustrate how the RB’s stability is affected, have been drawn. According to Lyapunov’s description, the symmetry of the closed-phase curves around any of its axes exhibit periodicity behavior through these near curves. The motion of the body is demonstrated, along with the beneficial consequences of the forces and moments imposed. Mastering the solutions to the angular velocities and Euler angles equations is vital for spacecraft motion and operations, as they play a crucial role in attitude determination and control. The angular velocity equations track changes in the spacecraft’s orientation over time, enabling precise adjustments to its trajectory. On the other hand, these angles provide a means to represent the spacecraft’s orientation in the 3D space, aiding in the calculation of maneuvers needed to achieve desired positions or orientations. Furthermore, their accurate solutions are essential for tasks like satellite stabilization, docking procedures, and navigation in space, ensuring that spacecraft can stay properly aligned for communication, scientific observations, and crucial mission operations.

## Description of the problem

**Fig. 1:**
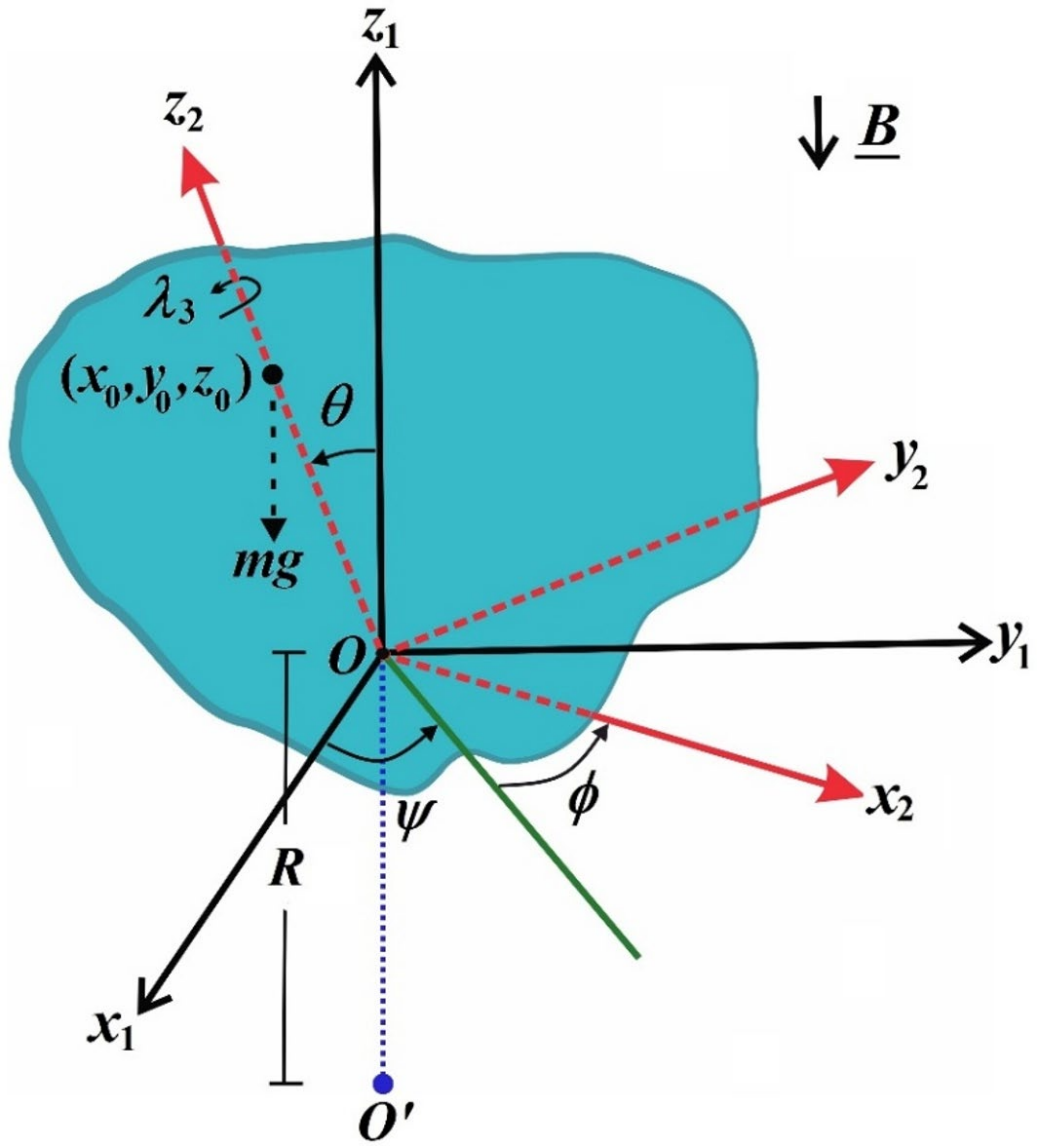
The dynamic structure of the RB.

This section presents an overall viewpoint of the studied problem where the 3D rotary movement of a heavy charged asymmetric RB of mass *m* around a fixed point *O* is examined. This motion is studied under the action of GT $$\underset{\raise0.3em\hbox{$\smash{\scriptscriptstyle-}$}}{\lambda } =({\lambda _1},{\lambda _2},{\lambda _3});\,\,{\lambda _1}={\lambda _2}=0$$, NFF emerging from a located-attracting center at a point $$O^{\prime}$$ on the opposite direction of the fixed axis $$O{z_1}$$ of a space-fixed frame $$O{x_1}{y_1}{z_1}$$, and a homogenous MF $$\underset{\raise0.3em\hbox{$\smash{\scriptscriptstyle-}$}}{B}$$ and the value of $$\delta$$ is defined in^21^ in details. Therefore, a moving frame called $$O{x_2}{y_2}{z_2}$$ rotates with the body is considered while being fixed inside it, as seen in Fig. 1.

The first three governing EOMs for the RB are given by Euler’s dynamics equation in the scalar form, the first term in the right-hand side for a uniform gravitational field $$mg\underline {{\hat {\kappa }}} \times \underset{\raise0.3em\hbox{$\smash{\scriptscriptstyle-}$}}{r}$$^1^, the second term for the MF impact $$\underset{\raise0.3em\hbox{$\smash{\scriptscriptstyle-}$}}{\sigma } \times \delta D\underline {{\hat {\kappa }}}$$^21^, and the NFF $$\Gamma (D\underline {{\hat {\kappa }}} \times \underline {{\hat {\kappa }}} )$$^22^. Therefore, we have

1 $$\begin{gathered} {D_1}\frac{{dp}}{{dt}}+({D_3} - {D_2}){\text{ }}qr+q{\lambda _3}=mg({z_0}\beta - {y_0}\gamma ) - \delta ({D_3}q\gamma - {D_2}r\beta )+\Gamma ({D_3} - {D_2})\gamma \beta , \hfill \\ {D_2}\frac{{dq}}{{dt}}+({D_1} - {D_3}){\text{ }}rp - p{\lambda _3}=mg({x_0}\gamma - {z_0}\alpha ) - \delta ({D_1}r\alpha - {D_3}p\gamma )+\Gamma ({D_1} - {D_3})\alpha \gamma , \hfill \\ {D_3}\frac{{dr}}{{dt}}+({D_2} - {D_1})pq=mg({y_0}\alpha - {x_0}\beta ) - \delta ({D_2}p\beta - {D_1}q\alpha )+\Gamma ({D_2} - {D_1})\alpha \beta . \hfill \\ \end{gathered}$$

The other three are given according to Poisson’s equation as^2^

2 $$\frac{{d\alpha }}{{dt}}=r\beta - q\gamma ,{\text{ }}\,\,\,\,\,\,\,\,\frac{{d\beta }}{{dt}}=p\gamma - r\alpha ,{\text{ }}\,\,\,\,\,\,\,\,\,{\text{ }}\frac{{d\gamma }}{{dt}}=q\alpha - p\beta .$$

It is important to keep in mind that, Eqs. (1) and (2) are the fundamental equations for the RB’s rotatory movement. Then, their related first integrals have the below forms^43^

3 $$\begin{gathered} {D_1}{p^2}+{D_2}{q^2}+{D_3}{r^2}+2mg({x_0}\alpha +{y_0}\beta +{z_0}\gamma )+\Gamma ({D_1}{\alpha ^2}+{D_2}{\beta ^2}+{D_3}{\gamma ^2})={D_1}p_{0}^{2}\, \hfill \\ \quad \,\,\,\,\,\,\,\,\,\,\,\,\,\,\,\,\,\,\,\,\,+{D_2}q_{0}^{2}+{D_3}r_{0}^{2}+2mg({x_0}{\alpha _0}+{y_0}{\beta _0}+{z_0}{\gamma _0})+\Gamma ({D_1}\alpha _{0}^{2}+{D_2}\beta _{0}^{2}+{D_3}\gamma _{0}^{2}), \hfill \\ {D_1}p\alpha +{D_2}q\beta +({D_3}r+{\lambda _3})\gamma +\frac{\delta }{2}({D_1}{\alpha ^2}+{D_2}{\beta ^2}+{D_3}{\gamma ^2})={D_1}{p_0}{\alpha _0}+{D_2}{q_0}{\beta _0} \hfill \\ \,\,\,\,\,\,\,\,\,\,\,\,\,\,\,\,\,\,\,\,\,\,\,\,\,\,\,\,\,+({D_3}{r_0}+{\lambda _3}){\gamma _0}+\frac{\delta }{2}({D_1}\alpha _{0}^{2}+{D_2}\beta _{0}^{2}+{D_3}\gamma _{0}^{2}), \hfill \\ {\alpha ^2}+{\beta ^2}+{\gamma ^2}=1, \hfill \\ \end{gathered}$$

whereas $({p_0},{q_0},{r_0})$ and $({\alpha _0},{\beta _0},{\gamma _0})$ are the initial values that correspond to the body’s angular velocity and the unit vector in $O{z_1}$ direction, respectively.

Simplifying the analysis process is a crucial step. Then, one can consider the following parameters and variables

4 $$\begin{gathered} {D_{11}}=\frac{{{D_3} - {D_2}}}{{{D_1}}},{\text{ }}\,{D_{21}}=\frac{{{D_1} - {D_3}}}{{{D_2}}},{\text{ }}\,{D_{31}}=\frac{{{D_2} - {D_1}}}{{{D_3}}},\,\,\,\,{D_4}=\frac{{{D_1}}}{{{D_3}}},{\text{ }}\,{D_5}=\frac{{{D_2}}}{{{D_3}}},\,\,\,{\rho ^2}=\frac{{mg\ell }}{{{D_3}}}, \hfill \\ {\ell ^2}=x_{0}^{2}+y_{0}^{2}+z_{0}^{2},\,\,\,\,\,\tau ={r_0}t,\,\,\,\,\,{x_0}=\ell {{x^{\prime}}_0},{\text{ }}\,\,{y_0}=\ell {{y^{\prime}}_0},{\text{ }}\,\,{z_0}=\ell {{z^{\prime}}_0},\,\,\,\,\upsilon =\frac{{\Gamma {\gamma _0}}}{{{c^2}}},\,\,\,{p_1}=\frac{p}{{\rho \sqrt {{\gamma _0}} }}, \hfill \\ {q_1}=\frac{q}{{\rho \sqrt {{\gamma _0}} }},{\text{ }}\,\,{r_1}=\frac{r}{{{r_0}}},\,\,\,\,{\alpha _1}=\frac{\alpha }{{{\gamma _0}}},{\text{ }}\,\,{\beta _1}=\frac{\beta }{{{\gamma _0}}},{\text{ }}\,\,{\gamma _1}=\frac{\gamma }{{{\gamma _0}}},\,\,\,\,\varepsilon =\frac{{\rho \sqrt {{\gamma _0}} }}{{{r_0}}},{\text{ }} \hfill \\ \end{gathered}$$

where $\varepsilon$ is a small parameter defining the dimensionless of the system and related to the high value of ${r_0}$.

Making use of (4) into (1), (2), and (3), yields

5 $$\begin{gathered} {{\dot {p}}_1}+({D_{11}}{r_1}+\frac{{{\lambda _3}}}{{{D_1}{r_0}}}){q_1}=\varepsilon \{ \frac{{{{z^{\prime}}_0}{\beta _1} - {{y^{\prime}}_0}{\gamma _1}}}{{{D_4}}} - \frac{{\delta {r_0}}}{{{D_1}{\rho ^2}}}(\varepsilon {D_3}{q_1}{\gamma _1} - {D_2}{r_1}{\beta _1})+\upsilon {D_{11}}{\beta _1}{\gamma _1}\} , \hfill \\ {{\dot {q}}_1}+({D_{21}}{r_1} - \frac{{{\lambda _3}}}{{{D_2}{r_0}}}){p_1}=\varepsilon \{ \frac{{{{x^{\prime}}_0}{\gamma _1} - {{z^{\prime}}_0}{\alpha _1}}}{{{D_5}}} - \frac{{\delta {r_0}}}{{{D_2}{\rho ^2}}}({D_1}{r_1}{\alpha _1} - \varepsilon {D_3}{p_1}{\gamma _1})+\upsilon {D_{21}}{\alpha _1}{\gamma _1}\} , \hfill \\ {{\dot {r}}_1}{\text{+ }}{\varepsilon ^2}{D_{31}}{p_1}{q_1}={\text{ }}{\varepsilon ^2}\{ {{y^{\prime}}_0}{\alpha _1} - {{x^{\prime}}_0}{\beta _1} - \frac{{\varepsilon \delta {r_0}}}{{{D_3}{\rho ^2}}}(\frac{{{p_1}{\beta _1}}}{{{D_5}}} - \frac{{{q_1}{\alpha _1}}}{{{D_4}}})+\upsilon {D_{31}}{\alpha _1}{\beta _1}\} , \hfill \\ {{\dot {\alpha }}_1}={r_1}{\beta _1} - \varepsilon {q_1}{\gamma _1},\quad \quad \quad {{\dot {\beta }}_1}=\varepsilon {p_1}{\gamma _1} - {r_1}{\alpha _1},\quad \quad \quad {{\dot {\gamma }}_1}=\varepsilon ({q_1}{\alpha _1} - {p_1}{\beta _1}), \hfill \\ \end{gathered}$$

6 $$r_{1}^{2}=1+{\varepsilon ^2}{K_1},{\text{ }}\,\,\,\,\,{r_1}{\gamma _1}=1+\varepsilon {K_2},{\text{ }}\,\,\,\,\alpha _{1}^{2}+\beta _{1}^{2}+\gamma _{1}^{2}=\gamma _{0}^{{ - 2}},$$

where

7 $$\begin{gathered} {K_1}={D_4}(p_{{10}}^{2} - p_{1}^{2})+{D_5}(q_{{10}}^{2} - q_{1}^{2})+2[{{x^{\prime}}_0}({\alpha _{10}} - {\alpha _1})+{{y^{\prime}}_0}({\beta _{10}} - {\beta _1})+{{z^{\prime}}_0}(1 - {\gamma _1})] \hfill \\ {\text{ }}\,\,\,\,\,\,\,\,\,\,\,\,\,\,\,\,\,\,\,\,\,\,\,\,\,\,\,\,\,+\upsilon [{D_4}(\alpha _{{10}}^{2} - \alpha _{1}^{2})+{D_5}(\beta _{{10}}^{2} - \beta _{1}^{2})+(1 - \gamma _{1}^{2})], \hfill \\ {K_2}={D_4}({p_{10}}{\alpha _{10}} - {p_1}{\alpha _1})+{D_5}({q_{10}}{\beta _{10}} - {q_1}{\beta _1})+\frac{{{\lambda _3}(1 - {\gamma _1})}}{{{D_3}\rho \sqrt {{\gamma _0}} {\text{ }}}}+\frac{{\delta \sqrt {{\gamma _0}} }}{{2\rho }}[{D_4}(\alpha _{{10}}^{2} - \alpha _{1}^{2}) \hfill \\ \,\,\,\,\,\,\,\,\,\,\,\,\,\,\,\,\,\,\,\,\,\,\,\,\,\,\,\,\,\,\,\,\,\,+{D_5}(\beta _{{10}}^{2} - \beta _{1}^{2})+(1 - \gamma _{1}^{2})], \hfill \\ \end{gathered}$$

and the differentiation with respect to $\tau$ is represented by the dot.

In accordance with integrals (6), we can easily obtain ${r_1}$ and ${\gamma _1}$ formulae as

8 $${r_1}=1+{\varepsilon ^2}\frac{{{K_{11}}}}{2}+ \cdots ,\,\,\,\,\,\,\,\,\,\,\,\,\,\,\,\,\,\,\,\,\,\,\,\,\,\,\,\,\,\,\,\,\,\,\,\,\,\,\,\,\,{\gamma _1}=1+\varepsilon {K_{21}}+{\varepsilon ^2}({K_{22}} - \frac{{{K_{11}}}}{2})+ \cdots .$$

The system (5) and its integrals (6) will now be boiled down to a suitable pair of equations and a single first integral. To achieve this purpose, substituting (8) into (5) and (6) to approach the desired system and integral, as

9 $$\begin{gathered} {{\ddot {p}}_2}+{\mu ^2}{p_2}=\varepsilon {U_1}({p_2},{{\dot {p}}_2},{\alpha _2},{{\dot {\alpha }}_2},\varepsilon )+{\varepsilon ^2}{U_2}({p_2},{{\dot {p}}_2},{\alpha _2},{{\dot {\alpha }}_2},\varepsilon ), \hfill \\ {{\ddot {\alpha }}_2}+{\alpha _2}=\varepsilon {U_3}({p_2},{{\dot {p}}_2},{\alpha _2},{{\dot {\alpha }}_2},\varepsilon )+{\varepsilon ^2}{U_4}({p_2},{{\dot {p}}_2},{\alpha _2},{{\dot {\alpha }}_2},\varepsilon ), \hfill \\ \end{gathered}$$

and

10 $$\begin{gathered} \alpha _{2}^{2}+\dot {\alpha }_{2}^{2}+2\varepsilon [{\Lambda _1}{\alpha _2}{p_2}+{{\dot {\alpha }}_2}({\Lambda _2}{{\dot {p}}_2}+{K_{21}})]+{\varepsilon ^2}[\Lambda _{1}^{2}p_{2}^{2}+\Lambda _{2}^{2}\dot {p}_{2}^{2} - 2N{{\dot {\alpha }}_2}({\xi _2}{{\dot {\alpha }}_2} \hfill \\ \,\,\,\,\,\,\,\,\,\,\,\,\,\,\,\,\,+{K_{21}}{{\dot {p}}_2}+\frac{{{{y^{\prime}}_0}}}{{{D_4}}})+K_{{21}}^{2}+2{K_{22}} - {K_{11}}]=\frac{{1 - \gamma _{0}^{2}}}{{\gamma _{0}^{2}}}, \hfill \\ \end{gathered}$$

where

11 $${p_1}={p_2}+\varepsilon ({\xi _1}+{\xi _2}{\alpha _2}),\quad \quad \quad \quad \;\;\;\;\quad \,\,\,\,\,\,\,\,\,\,\,\,\,\,\,\,\,{\alpha _1}={\alpha _2}+\varepsilon {\Lambda _1}{p_2},$$

12 $$\begin{gathered} {q_1}=N\{ - {{\dot {p}}_2}+\varepsilon (\frac{{{{y^{\prime}}_0}}}{{{D_4}}} - {\xi _2}{{\dot {\alpha }}_2})\} +{\varepsilon ^2}\{ N{{\dot {p}}_2}[{\Lambda _1}(\frac{{\delta {D_2}}}{{{D_1}}}+\frac{{{{z^{\prime}}_0}}}{{{D_4}}})+{K_{11}}] - \frac{{{K_{11}}}}{{2{D_1}}}{{\dot {p}}_2}+N(\upsilon {D_{11}}{{\dot {\alpha }}_2} \hfill \\ \,\,\,\,\,\,\,\,\,\,\,\,\,\,\,\,\,\,\,\,\,\,\,\,\,\,\, - \frac{{{{y^{\prime}}_0}}}{{{D_4}}}{K_{21}})+\frac{{\delta {D_3}}}{{D_{1}^{2}}}({K_{21}} - {K_{11}})\} + \cdots , \hfill \\ {\beta _1}={{\dot {\alpha }}_2}+\varepsilon {\Lambda _2}{{\dot {p}}_2}+{\varepsilon ^2}[N(\frac{{{{y^{\prime}}_0}}}{{{D_4}}} - {\xi _2}{{\dot {\alpha }}_2} - {K_{21}}{{\dot {p}}_2}) - \frac{{{K_{11}}}}{2}{{\dot {\alpha }}_2}], \hfill \\ \end{gathered}$$

13 $$\begin{gathered} {U_1}=\frac{{N({D_{31}}{D_3}+D_{{11}}^{2})}}{{{D_{11}}{D_3}}}{p_2}{{\dot {p}}_2},\,\,\,\,\,\,\,\,\,\,\,\,\,\,\,\,\,\,\,\,\,\,\,\,\,\,\,\,\,\,\,\,\,\,\,\,\,\,\,\,{U_2}={u_1} - {\Lambda _1}{\xi _1}{p_2}(1 - {\mu ^2}), \hfill \\ {U_3}=0,\,\,\,\,\,\,\,\,\,\,\,\,\,\,\,\,\,\,\,\,\,\,\,\,\,\,\,\,\,\,\,\,\,\,\,\,\,\,\,\,\,\,\,\,\,\,\,\,\,\,\,\,\,\,\,\,\,\,\,\,\,\,\,\,\,\,\,\,\,\,\,\,\,\,{U_4}={u_2}+{\Lambda _1}(1 - {\mu ^2})(\xi +{\xi _1}{\alpha _2}), \hfill \\ \end{gathered}$$

and

14 $$\begin{gathered} {\mu ^2}={\chi ^2}+\frac{{{\lambda _3}({D_{11}}{D_1} - {D_2}{D_{21}})}}{{{D_2}{D_1}{r_0}}},{\text{ }}\,{\chi ^2}= - {D_{11}}{D_{21}},{\text{ }}\,N=\frac{{{D_1}{D_{11}}{r_0} - {\lambda _3}}}{{{D_1}D_{{11}}^{2}{r_0}}},{\text{ }}{\Lambda _2}={\Lambda _1} - N,{\text{ }} \hfill \\ \xi =\frac{{{{x^{\prime}}_0}({D_{11}}{D_1}{r_0}+{\lambda _3})}}{{{\mu ^2}{D_5}{D_1}{r_0}}},\,\,\,\,\,\,{\text{ }}\,\,\,\,\,{\xi _2}={\xi _1}+\frac{{\delta {D_4}{D_2} - {{z^{\prime}}_0}{D_1}}}{{{D_4}{D_1}}},\,\,\,\,\,\,\,\,\,\,\,\,{\Lambda _1}=\frac{{1+{D_{21}} - {{({D_2}{r_0})}^{ - 1}}{\lambda _3}}}{{1 - {\mu ^2}}}, \hfill \\ {\xi _1}=\frac{{{{z^{\prime}}_0}}}{{1 - {\mu ^2}}}(\frac{{{\lambda _3}}}{{{D_5}{r_0}{D_1}}} - \frac{{{D_5} - {D_4}{D_{11}}}}{{{D_4}{D_5}}})+\frac{\delta }{{1 - {\mu ^2}}}(\frac{{{D_{11}}D_{1}^{2} - D_{2}^{2}}}{{{D_2}{D_1}}}+\frac{{\delta {\lambda _3}}}{{{r_0}{D_2}}}),\,\,\,\,\,\,\,R=\frac{{{\lambda _3}}}{{{D_3}\rho \sqrt {{\gamma _0}} }}, \hfill \\ {K_{11}}={D_4}(p_{{20}}^{2} - p_{2}^{2})+{D_5}{N^2}(q_{{20}}^{2} - q_{2}^{2})+2[{{x^{\prime}}_0}({\alpha _{20}} - {\alpha _2})+{{y^{\prime}}_0}({{\dot {\alpha }}_{20}} - {{\dot {\alpha }}_2})]+\upsilon [{D_4}\;(\alpha _{{20}}^{2} \hfill \\ \quad \;\;\,\,\,\,\,\,\,\,\,\,\,\,\,\,\,\,\,\,\,\, - \alpha _{2}^{2})+{D_5}(\dot {\alpha }_{{20}}^{2} - \dot {\alpha }_{2}^{2})], \hfill \\ {K_{12}}={D_4}[\xi ({p_{20}} - {p_2})+{\xi _1}({p_{20}}{\alpha _{20}} - {p_2}{\alpha _2})] - \frac{{{D_5}{N^2}{{y^{\prime}}_0}{\xi _2}}}{{{D_4}}}({{\dot {p}}_{20}} - {{\dot {p}}_2})({{\dot {p}}_{20}}{{\dot {\alpha }}_{20}} - {{\dot {p}}_2}{{\dot {\alpha }}_2}) \hfill \\ \quad \;{\kern 1pt} \,\,\,\,+{{x^{\prime}}_0}{\Lambda _1}({p_{20}} - {p_2})+{{y^{\prime}}_0}{\Lambda _2}({{\dot {p}}_{20}} - {{\dot {p}}_2}) - ({{z^{\prime}}_0}+\upsilon ){K_{21}}+\upsilon [a{\Lambda _1}({p_{20}}{\alpha _{20}} - {p_2}{\alpha _2}) \hfill \\ \quad \;{\kern 1pt} {\kern 1pt} \,\,\,\,+{D_5}{\Lambda _2}({{\dot {p}}_{20}}{{\dot {\alpha }}_{20}} - {{\dot {p}}_2}{{\dot {\alpha }}_2})], \hfill \\ {K_{21}}={D_4}({p_{20}}{\alpha _{20}} - {p_2}{\alpha _2}) - {D_5}N({{\dot {p}}_{20}}{{\dot {\alpha }}_{20}} - {{\dot {p}}_2}{{\dot {\alpha }}_2}), \hfill \\ {K_{22}}={D_4}[{\Lambda _1}(p_{{20}}^{2} - p_{2}^{2})+\xi ({\alpha _{20}} - {\alpha _2})+{\xi _1}\;(\alpha _{{20}}^{2} - \alpha _{2}^{2})] - R{K_{21}} - {D_5}N[{\Lambda _2}(\dot {p}_{{20}}^{2} - \dot {p}_{2}^{2}) \hfill \\ \quad \;\;\,\,\,\,\,\,\,\,\,\,\, - \frac{{{{y^{\prime}}_0}}}{{{D_4}}}({{\dot {\alpha }}_{20}} - {{\dot {\alpha }}_2})+{\xi _2}(\dot {\alpha }_{{20}}^{2} - \dot {\alpha }_{2}^{2})]+\delta [{D_4}(\alpha _{{20}}^{2} - \alpha _{2}^{2})+{D_5}(\dot {\alpha }_{{20}}^{2} - \dot {\alpha }_{2}^{2})], \hfill \\ \end{gathered}$$

with

15 $$\begin{gathered} {u_1}=2\frac{{{D_{11}}{N^2}{{\dot {p}}_2}}}{{{D_3}{r_0}}}(\frac{{{{y^{\prime}}_0}}}{{{D_4}}}+{\xi _2}{{\dot {\alpha }}_2}) - {{\dot {p}}_2}N[{D_{11}}{{x^{\prime}}_0}{{\dot {\alpha }}_2} - {{y^{\prime}}_0}{\alpha _2}\frac{{{D_{11}}{D_4}+1}}{{{D_4}}}]+{p_2}\{ {D_{11}}{D_{31}}{N^2}[\dot {p}_{2}^{2} \hfill \\ \,\,\,\,\,\,\,\,\,+2{{\dot {p}}_2}(\frac{{{{y^{\prime}}_0}}}{{{D_4}}} - {\xi _2}{{\dot {\alpha }}_2})] - {\chi ^2}{K_{11}}+{D_{11}}{D_2}{K_{11}}+\frac{{{{y^{\prime}}_0}{{\dot {\alpha }}_2}}}{{{D_4}}}+\delta [\frac{{{D_{11}}{D_3}}}{{{D_2}}}+\frac{{{D_2}({D_3}+1)}}{{{D_1}}}]+\frac{{{\lambda _3}}}{{2{r_0}}} \hfill \\ \,\,\,\,\,\,\,\, \times {K_{11}}\frac{{{D_{21}}{D_2} - {D_1}{D_{11}}}}{{{D_1}{D_2}}}\} +2\frac{{{{x^{\prime}}_0}{K_{21}}{D_{11}}}}{{{D_5}}}+\frac{{{\lambda _3}}}{{{D_1}{r_0}}}[\frac{{{{x^{\prime}}_0}{K_{21}}}}{{{D_5}}} - (\frac{{{{z^{\prime}}_0}}}{{{D_5}}}+\upsilon {D_{21}})]+\frac{{{{z^{\prime}}_0}{\alpha _2}}}{{{D_4}}}+\upsilon {D_{11}} \hfill \\ \,\,\,\,\,\,\,\,\, \times \{ (\frac{{{\mu ^2}}}{{{D_{11}}}} - 1)+{p_2}[1 - {{\dot {\alpha }}_2}({{\dot {\alpha }}_2} - 2{\Lambda _2}{{\dot {p}}_2})] - {\alpha _2}[N{{\dot {\alpha }}_2}{{\dot {p}}_2}(1 - {D_{31}})+{K_{21}}(1+{K_{21}})]\} , \hfill \\ \end{gathered}$$

16 $$\begin{gathered} {u_2}={{\dot {\alpha }}_2}[{{y^{\prime}}_0}{\alpha _2} - {{x^{\prime}}_0}{{\dot {\alpha }}_2} - N{p_2}{{\dot {p}}_2}(1 - {D_{31}})] - \frac{{{{x^{\prime}}_0} - {{z^{\prime}}_0}{\alpha _2}}}{{{D_5}}}+\frac{{{\lambda _3}{p_2}{K_{22}}}}{{{D_2}{r_0}}} - {\alpha _2}\{ {K_{11}} - \frac{{\delta {D_1}}}{{{D_2}}} \hfill \\ \quad \;{\kern 1pt} \,\,\,\,\,\,\,\,+{N^2}\dot {p}_{2}^{2} - \upsilon [\dot {\alpha }_{2}^{2} - {D_{21}}(2{K_{21}}+1)]\} . \hfill \\ \end{gathered}$$

In light of the above description, the $O{x_2}$and $O{z_2}$are chosen to prevent an obtuse angle with the ${z_1}$-axis. Mathematically, this can be interpreted as

17 $${\alpha _{\text{0}}} \geqslant {\text{0,}}\quad \quad \quad \quad \,\,\,\,\,\,\,\,\,\,\,\,\,\,\,\,\,\,\,\,\,\,\,\,\,\,\,\,{\text{0}}\;{\text{<}}{\gamma _{\text{0}}}{\text{<}}\,{\text{1}}{\text{.}}$$

One must note that in some restricted circumstances, such as in the absence of NFF, GT, and MF, one can obtain some limited cases^21,22^ from this study.

It must be mentioned that reducing the six equations of motion and the three initial integrals for the examined model to two equations and one integral has several advantages, as it leads to a simpler mathematical analysis, in which the researchers and engineers benefit from the ability to examine the system more thoroughly with fewer equations. With fewer equations, the computational load is reduced, resulting in quicker and more efficient computations, which aid in a better comprehension of the fundamental physical phenomena governing the system’s motion. This procedure can reveal deeper insights into variable interactions. At times, it enhances the accuracy of approximate solutions, thereby improving the quality of models and simulations.

## Proceeding with the periodic solution

This section seeks to clarify how we can apply the approach of KBM, to generate the periodic or quasi-periodic solutions of systems (9), (11) and (12), where the constraint ${D_1}<{D_2}<{D_3}$ or ${D_1}>{D_2}>{D_3}$ is considered when examining an instance of nonzero basic amplitudes. The mentioned method has an advantage over Poincaré’s method of small parameter in that the dependence of the solutions on the independent periodicity conditions need not be specified a priori^49^. The criterion of elimination of secular producing terms introduced for the determination of the arbitrary functions appear in the expressions in this paper. PMSP requires the convergence of series in a small parameter, which represent periodic solutions, but in the description of the KBM method it is emphasized that the question of the convergence of small parameter expansions does not arise at all^50^. The generating system of (9) can be obtained at $\varepsilon =0$as follows

18 $$\ddot {p}_{2}^{{(0)}}+{\mu ^2}p_{2}^{{(0)}}=0,{\text{ }}\quad \quad {\text{ }}\,\,\,\,\,\,\,\,\,\,\,\,\,\,\,\,\,\,\,\,\,\,\,\,\,\,\,\,\,\,\,\,\,\,\,\,\,\,\,\,\,\,\,\,\ddot {\alpha }_{2}^{{(0)}}+\alpha _{2}^{{(0)}}=0.$$

It is noted that this first equation has the frequency $\mu$ and while 1 is the frequency of the second one. According to system (9), one can observe that three possible trials of the frequencies can be examined, namely, to be different and commensurate, equal, or not commensurate. Then, the first scenario of different and commensurable frequencies is taken into consideration. Thus, the aforementioned system has solutions with period ${T_0}=2\pi n$, as follows

19 $$p_{2}^{{(0)}}(\tau )=F\cos (\mu \tau - \zeta ),{\text{ }}\quad \alpha _{2}^{{(0)}}(\tau )={H_3}\cos \tau ,$$

in which $F=\sqrt {H_{1}^{2}+H_{2}^{2}} ,{\text{ }}\zeta ={\tan ^{ - 1}}\frac{{{H_2}}}{{{H_1}}},$ and ${H_a}\,\,(a=1,2,3)$ are constants. We can infer from these solutions that system (9) has periodic solutions with a period ${T_0}+\Re$, where the parameter $\Re$ can be presented as a function of $\varepsilon$, i.e. $\Re =\Re (\varepsilon )$. For the case $\Re (0)=0$, the system’s time period reduces to a corresponding one of the system (18). After absorbing and thoroughly inspecting the previous analysis, it is possible to illustrate the initial conditions of the desired solutions as

20 $${p_2}(0,\varepsilon )={\tilde {H}_1},{\text{ }}\,\,\,\,\,\,\,{\dot {p}_2}(0,\varepsilon )=\mu {\tilde {H}_2},{\text{ }}\,\,\,\,\,\,\,\,\,\,\,{\alpha _2}(0,\varepsilon )={\tilde {H}_3},{\text{ }}\,\,\,\,\,\,\,\,\,\,{\text{ }}{\dot {\alpha }_2}(0,\varepsilon )=0,$$

where

21 $${H_j} \to {\tilde {H}_a}={H_a}+{\varpi _a};\quad \quad \quad (a=1,2,3).$$

Here, ${\varpi _a}$ represent the variations in the values ${p_2},{\dot {p}_2},$ and ${\alpha _2}$ in (9) other than the values in (18) at $\tau =0$. Therefore, one can write ${\varpi _a}={\varpi _a}(\varepsilon )$ as ${\varpi _a}(0)=0$ is satisfied. The values of ${H_3}$ and ${\varpi _3}$ can calculated by using the integral (10) and the criteria (20) at $\tau =0$, as

22 $$0<{\tilde {H}_3}={{\sqrt {\,1 - \gamma _{0}^{2}} } \mathord{\left/ {\vphantom {{\sqrt {\,1 - \gamma _{0}^{2}} } {{\gamma _0}}}} \right. \kern-0pt} {{\gamma _0}}},\,\,\,\,\,{\varpi _3}= - \varepsilon \,{\Lambda _1}{\tilde {H}_1}+ \cdots \;.$$

This indicates that the desired solutions will take the forms

23 $$\begin{gathered} {p_2}=\tilde {F}\cos ({\Omega _1} - \eta )+\sum\limits_{{n=1}}^{d} {{\varepsilon ^n}} {{\tilde {p}}_n}(\tilde {F},{\Omega _1})+O({\varepsilon ^{d+1}}), \hfill \\ {\alpha _2}={{\tilde {H}}_3}\cos {\Omega _2}+\sum\limits_{{n=1}}^{d} {{\varepsilon ^n}} {{\tilde {\alpha }}_n}(\tilde {F},{\Omega _2})+O({\varepsilon ^{d+1}}), \hfill \\ \end{gathered}$$

where ${\tilde {p}_n}$ and ${\tilde {\alpha }_n}$ represent, respectively, periodic functions of ${\Omega _l}\,\,(l=1,2)$.

The derivatives of $\tilde {F},\,$and ${\Omega _l}$, can be expressed according to the procedure of the KBM method as^21^

24 $$\begin{gathered} \frac{{d\tilde {F}}}{{d\tau }}=\sum\limits_{{n=1}}^{d} {{\varepsilon ^n}} {{\tilde {F}}_n}(\tilde {F})+O({\varepsilon ^{d+1}}), \hfill \\ \frac{{d{\Omega _1}}}{{d\tau }}=\mu +\sum\limits_{{n=1}}^{d} {{\varepsilon ^n}} {\Omega _{1n}}(\tilde {F})+O({\varepsilon ^{d+1}}), \hfill \\ \frac{{d{\Omega _2}}}{{d\tau }}=1+\sum\limits_{{n=1}}^{d} {{\varepsilon ^n}} {\Omega _{2n}}(\tilde {F})+O({\varepsilon ^{d+1}}). \hfill \\ \end{gathered}$$

Estimation of the functions ${\dot {p}_2},\,{\dot {\alpha }_2},\,{\ddot {p}_2},$ and ${\ddot {\alpha }_2},$ can be obtained as

25 $$\begin{gathered} {{\dot {p}}_2}=\frac{{d\tilde {F}}}{{d\tau }}\frac{{\partial {p_2}}}{{\partial \tilde {F}}}+\frac{{d{\Omega _1}}}{{d\tau }}\frac{{\partial {p_2}}}{{\partial {\Omega _1}}},{\text{ }}\quad \quad \quad \quad {\text{ }}\,\,\,\,\,\,\,\,\,\,\,\,\,\,\,\,\,\,\,\,\,\,\,{{\dot {\alpha }}_2}=\frac{{d\tilde {F}}}{{d\tau }}\frac{{\partial {\alpha _2}}}{{\partial \tilde {F}}}+\frac{{d{\Omega _2}}}{{d\tau }}\frac{{\partial {\alpha _2}}}{{\partial {\Omega _2}}}, \hfill \\ {{\ddot {p}}_2}={(\frac{{d\tilde {F}}}{{d\tau }})^2}\frac{{{\partial ^2}{p_2}}}{{\partial {{\tilde {F}}^2}}}+\frac{{{d^2}\tilde {F}}}{{d{\tau ^2}}}\frac{{\partial {p_2}}}{{\partial \tilde {F}}}+2\frac{{d\tilde {F}}}{{d\tau }}\frac{{d{\Omega _1}}}{{d\tau }}\frac{{{\partial ^2}{p_2}}}{{\partial \tilde {F}\partial {\Omega _1}}}+{(\frac{{d{\Omega _1}}}{{d\tau }})^2}\frac{{{\partial ^2}{p_2}}}{{\partial \Omega _{1}^{2}}}+\frac{{{d^2}{\Omega _1}}}{{d{\tau ^2}}}\frac{{\partial {p_2}}}{{\partial {\Omega _1}}}, \hfill \\ {{\ddot {\alpha }}_2}={(\frac{{d\tilde {F}}}{{d\tau }})^2}\frac{{{\partial ^2}{\alpha _2}}}{{\partial {{\tilde {F}}^2}}}+\frac{{{d^2}\tilde {F}}}{{d{\tau ^2}}}\frac{{\partial {\alpha _2}}}{{\partial \tilde {F}}}+2\frac{{d\tilde {F}}}{{d\tau }}\frac{{d{\Omega _2}}}{{d\tau }}\frac{{{\partial ^2}{p_2}}}{{\partial \tilde {F}\partial {\Omega _2}}}+{(\frac{{d{\Omega _2}}}{{d\tau }})^2}\frac{{{\partial ^2}{\alpha _2}}}{{\partial \Omega _{2}^{2}}}+\frac{{{d^2}{\Omega _2}}}{{d{\tau ^2}}}\frac{{\partial {\alpha _2}}}{{\partial {\Omega _2}}}. \hfill \\ \end{gathered}$$

Substituting (23) and (25) into (14) to obtain

26 $$\begin{gathered} K_{{11}}^{{(0)}}={L_0}\sin {\Omega _1}\sin (2\zeta - {\Omega _1})+({L_1}+2{L_3})(1 - \cos {\Omega _2})+({L_2}+2{L_4})\sin {\Omega _2}, \hfill \\ K_{{21}}^{{(0)}}= - {L_5}\sin {\Omega _2}\sin (\zeta - {\Omega _1})+{L_6}[\cos \zeta - \cos {\Omega _2}\cos (\zeta - {\Omega _1})], \hfill \\ K_{{12}}^{{(0)}}=\,{L_7}[\cos \zeta - \cos (\zeta - {\Omega _1})]+{L_8}\sin {\Omega _2}\sin (\zeta - {\Omega _1}) - {L_9}[\cos \zeta - \cos (\zeta - {\Omega _1}) \hfill \\ \,\,\,\,\,\,\,\,\,\,\,\,\,\, \times \cos {\Omega _2}] - {L_{10}}[\sin \zeta +\sin (\zeta - {\Omega _1})]+{L_{11}}[\cos \zeta - \cos {\Omega _2}\cos (\zeta - {\Omega _1})] \hfill \\ \,\,\,\,\,\,\,\,\,\,\,\,+{L_{12}}\sin {\Omega _2}\sin (\zeta - {\Omega _1})+{L_{13}}[\cos \zeta - \cos (\zeta - {\Omega _1})] - {L_{14}}[\cos (\zeta - {\Omega _1}) \hfill \\ \,\,\,\,\,\,\,\,\,\,\,\,\,\, \times \cos {\Omega _2} - \cos \zeta ] - {L_{15}}\sin {\Omega _2}\sin (\zeta - {\Omega _1})[\sin \zeta +\sin (\zeta - {\Omega _1})], \hfill \\ K_{{22}}^{{(0)}}={L_{16}}(1 - \cos {\Omega _2})+{L_{17}}\sin {\Omega _2} - {L_{18}}[\cos \zeta - \cos {\Omega _2}\cos (\zeta - {\Omega _1})]+{L_{19}}\sin {\Omega _2} \hfill \\ \,\,\,\,\,\,\,\,\,\,\,\,\,\, \times \sin (\zeta - {\Omega _1})+{L_{20}}\sin {\Omega _2} - {L_{21}}\sin {\Omega _1}\sin (2\zeta - {\Omega _1})+{L_{22}}\sin \Omega _{2}^{2}. \hfill \\ \end{gathered}$$

where the constants ${L_\Delta },(\Delta =0,1, \ldots ,22)$ are derived in Appendix A.

Substituting (23) and (25) into (13), one can estimate the functions $U_{b}^{{{\text{(0)}}}}\,\,\,(b=1,2,3,4{\text{)}}$as follows

27 $$\begin{gathered} U_{1}^{{(0)}} = \frac{{\tilde{F}^{2} N(D_{{11}}^{2} + D_{3} D_{{31}} )}}{{D_{3} D_{{11}} }}\sin 2(\zeta - \Omega _{1} ),U_{2}^{{(0)}} = u_{1}^{{(0)}} + \tilde{F}\Lambda _{1} \xi _{2} \cos (\zeta - \Omega _{1} ), \hfill \\ U_{3}^{{(0)}} = 0,\quad U_{4}^{{(0)}} = u_{2}^{{(0)}} + \Lambda _{1} (1 - \mu ^{2} )[\xi + \tilde{H}_{3} \xi _{1} \cos \Omega _{2} ], \hfill \\ \end{gathered}$$

where

28 $$\begin{gathered} u_{1}^{{(0)}}={{\rm M}_0}+{{\rm M}_1}\cos (\zeta - {\Omega _1})+\cos {\Omega _2}\{ {{\rm M}_7}[{D_4}\cos {\Omega _2}\cos (\zeta - {\Omega _1}) - {D_4}\cos \zeta \hfill \\ \,\,\,\,\,\,\,\,\,\,+{D_5}N\sin (\zeta - {\Omega _1})\sin {\Omega _2}]+\,{{\rm M}_8}\sin {\Omega _2}\sin (\zeta - {\Omega _1})\} - \,{{\rm M}_2}\cos (\zeta - {\Omega _1})\cos {\Omega _2} \hfill \\ \,\,\,\,\,\,\,\,\,\, - {{\rm M}_2}\cos \zeta +{D_5}N{{\rm M}_2}\sin {\Omega _2}\sin (\zeta - {\Omega _1}) - {{\rm M}_6}\cos (\zeta - {\Omega _1})\sin {\Omega _2} - {{\rm M}_6} \hfill \\ \,\,\,\,\,\,\,\,\,\, \times \sin (\zeta - {\Omega _1})[N\cos {\Omega _2}+{{\rm M}_{12}}({{x^{\prime}}_0}\sin {\Omega _2}+{{y^{\prime}}_0}\cos {\Omega _2})]+\cos (\zeta - {\Omega _1})\{ {{\rm M}_9}{{\rm M}_3} \hfill \\ \,\,\,\,\,\,\,\,\,\, \times \sin {\Omega _1}\sin (2\zeta - {\Omega _1})+{{\rm M}_3}[\upsilon ({D_4}\cos {\Omega _2} - {D_5}\sin {\Omega _2} - {D_4})+2({{x^{\prime}}_0}\cos {\Omega _2} - {{x^{\prime}}_0} \hfill \\ \,\,\,\,\,\,\,\,\,\,+{{y^{\prime}}_0}\sin {\Omega _2})]\} - {{\rm M}_5}+{{\rm M}_{10}}\cos {\Omega _2}+\cos (\zeta - {\Omega _1})\{ {D_{11}} - {D_{11}}\tilde {H}_{3}^{2}{\sin ^2}{\Omega _2}+2{D_{11}} \hfill \\ \,\,\,\,\,\,\,\,\,\, \times {{\rm M}_{11}}\sin {\Omega _2}\sin (\zeta - {\Omega _1})\} +2{{\rm M}_4}\sin {(\zeta - {\Omega _1})^2} - 2{{\rm M}_{35}}\sin {(\zeta - {\Omega _1})^2}\sin {\Omega _2}, \hfill \\ \end{gathered}$$

29 $$\begin{gathered} u_{2}^{{(0)}}={{\rm M}_{13}}\cos {\Omega _2} - {{\rm M}_{16}}+{{\rm M}_{17}}\sin {\Omega _1}\sin (2\zeta - {\Omega _1})+{{\rm M}_{18}}\cos {\Omega _2}\sin {\Omega _1}\sin (2\zeta - {\Omega _1}) \hfill \\ \,\,\,\,\,\,\,\,\,\, - {{\rm M}_{19}}\cos {\Omega _2}\sin {\zeta ^2} - {{\rm M}_{20}}\cos {\Omega _2}\sin {(\zeta - {\Omega _1})^2} - {{\rm M}_{21}}\sin {\Omega _2}\sin 2(\zeta - {\Omega _1}) \hfill \\ \,\,\,\,\,\,\,\,\,\, - {{\rm M}_{22}}\cos {\Omega _2}+{{\rm M}_{23}}\cos {\Omega _2}[\cos {\Omega _2}\cos (\zeta - {\Omega _1}) - \cos \zeta ]+{{\rm M}_{24}}\sin (\zeta - {\Omega _1}) \hfill \\ \,\,\,\,\,\,\,\,\,\, \times \sin 2{\Omega _2}+{{\rm M}_{31}}\cos {\Omega _2}[{D_4}(\cos {\Omega _2} - 1) - {D_5}\sin {\Omega _2}]+{{\rm M}_{32}}(1+3\cos 2{\Omega _2} \hfill \\ \,\,\,\,\,\,\,\,\,\, - 4\cos {\Omega _2})+{{\rm M}_{33}}\sin 2{\Omega _2}(\upsilon {{\tilde {H}}_3}\sin {\Omega _2} - 3{{y^{\prime}}_0})+\,{{\rm M}_{34}}\cos {\Omega _2}+\cos (\zeta - {\Omega _1}) \hfill \\ \,\,\,\,\,\,\,\,\,\, \times \{ {{\rm M}_{25}}(1 - \cos {\Omega _2})+{{\rm M}_{26}}\sin {\Omega _2} - {{\rm M}_{27}}\cos \zeta +{{\rm M}_{28}}\cos {\Omega _2}\cos (\zeta - {\Omega _1})+{{\rm M}_{29}} \hfill \\ \,\,\,\,\,\,\,\,\,\, \times \sin {\Omega _2}\sin (\zeta - {\Omega _1})+{{\rm M}_{30}}\sin {\Omega _2} - {{\rm M}_{15}}\sin {\Omega _2}\sin (2\zeta - {\Omega _1})+{{\rm M}_{14}}\sin \Omega _{2}^{2}\} . \hfill \\ \end{gathered}$$

where the constant value ${{\rm M}_n},\,\,(n=0,1, \ldots ,35)$ are provided in Appendix B.

Consequently, adjusting the coefficients of the same powers of $\varepsilon$ in each side of Eq. (9), yields.

Coefficients of $$\varepsilon$$:

30 $$\begin{gathered} \frac{{{\partial ^2}{{\tilde {p}}_1}}}{{\partial \Omega _{1}^{2}}}+{{\tilde {p}}_1}=2{\mu ^{ - 1}}[\sin {\Omega _1}({{\tilde {F}}_1}\cos \zeta +\tilde {F}{\Omega _{11}}\sin \zeta )+\cos {\Omega _1}(\tilde {F}{\Omega _{11}}\cos \zeta - {{\tilde {F}}_1}\sin \zeta )] \hfill \\ \quad \quad \quad \quad \,\,\,\,\,\,\,\,\,\,\,\,+{Q_1}\sin (\zeta - {\Omega _1}), \hfill \\ \frac{{{\partial ^2}{{\tilde {\alpha }}_1}}}{{\partial \Omega _{2}^{2}}}+{{\tilde {\alpha }}_1}=2{{\tilde {H}}_3}{\Omega _{21}}\cos {\Omega _2}. \hfill \\ \end{gathered}$$

Removing secular terms^10^ from (30) produces

31 $${\tilde {F}_1}\cos \zeta +\tilde {F}{\Omega _{11}}\sin \zeta =0,{\text{ }}\tilde {F}{\Omega _{11}}\cos \zeta - {\tilde {F}_1}\sin \zeta ,{\text{ }}{\Omega _{21}}=0.$$

The equations in (31), is a system of linear equations in ${\tilde {F}_1}$ and ${\Omega _{11}}$ whose determinant is

32 $$\Delta =\tilde {F}\,\,\left| \begin{gathered} \,\cos \zeta \,\,\,\,\,\,\,\,\,\, - \sin \zeta \hfill \\ \,\sin \zeta \,\,\,\,\,\,\,\,\,\,\,\,\,\,\cos \zeta \, \hfill \\ \end{gathered} \right| \ne 0,$$

which can’t be satisfied unless the following is true

33 $${\tilde {F}_1}={\Omega _{11}}=0$$

In light of that, the solutions of equations (30) may be formed as

34 $$\begin{gathered} {{\tilde {p}}_1}={c_1}\cos {\Omega _1}+{c_2}\sin {\Omega _1} - {Q_2}\cos 2(\zeta - {\Omega _1}), \hfill \\ {{\tilde {\alpha }}_1}={c_3}\cos {\Omega _2}+{c_4}\sin {\Omega _2}. \hfill \\ \end{gathered}$$

Coefficients of $${\varepsilon ^2}$$:

35 $$\begin{gathered} \frac{{{\partial ^2}{{\tilde {p}}_2}}}{{\partial \Omega _{1}^{2}}}+{{\tilde {p}}_2}={\mu ^{ - 2}}u_{1}^{{(0)}}+{Q_3}\cos (\zeta - {\Omega _1}) - 2{\mu ^{ - 1}}[\sin {\Omega _1}({{\tilde {F}}_2}\cos \zeta +\tilde {F}{\Omega _{12}}\sin \zeta ) \hfill \\ \,\,\,\,\,\,\,\,\,\,\,\,\,\,\,\,\,\,\,\,\,\,\,\,\,\,\,\,\,\,\,\,\,\,\,\,\,\,\,\,\,+\cos {\Omega _1}(\tilde {F}{\Omega _{12}}\cos \zeta - {{\tilde {F}}_2}\sin \zeta )], \hfill \\ \frac{{{\partial ^2}{{\tilde {\alpha }}_2}}}{{\partial \Omega _{2}^{2}}}+{{\tilde {\alpha }}_2}=u_{2}^{{(0)}}+2{{\tilde {H}}_3}{\Omega _{22}}\cos {\Omega _2} - {Q_4} - {Q_5}\cos {\Omega _2}. \hfill \\ \end{gathered}$$

Using (28), (29), (33), and (35), and getting rid of terms that create secular ones, yields

36 $$\begin{gathered} {{\tilde {F}}_2}={(\tan {\zeta ^2} - 1)^{ - 1}}\{ {Q_6}{\sec ^2}\zeta \sin 2{\Omega _2} - {Q_7}\sec \zeta \sin (\zeta - {\Omega _2}) - ({Q_8}+{Q_{10}}){\sec ^2}\zeta \sin {\Omega _2} \hfill \\ \,\,\,\,\,\,\,\,\,\,\,\,\,\,\,\,\,\,\,\,\,\,\,\,\,\,\,\,\,\,\,\,\,\,\,\,\,\,\,\,\,\,\,+{Q_9}{\sec ^2}\zeta \cos {\Omega _2} - \,{Q_{10}}(\cos 2\zeta +\sin 2\zeta - 1)\sec \zeta \cos {\Omega _2}\} , \hfill \\ {\Omega _{12}}=\sec 2\zeta \{ {Q_{25}} - {Q_{16}}\cos \zeta \cos (\zeta +{\Omega _2}) - {Q_{19}}\cos {\Omega _2}(3\cos \zeta +\cos 3\zeta - 4\sin {\zeta ^3}) \hfill \\ \,\,\,\,\,\,\,\,\, - {Q_{25}}\cos {\Omega _2}+({Q_{30}} - {Q_{27}})(\cos {\Omega _2} - 1)+\,({Q_{26}}+{Q_{28}} - {Q_{31}})\sin {\Omega _2}+[{Q_{13}}+{Q_{17}} \hfill \\ \,\,\,\,\,\,\,\,\,+{Q_{22}}+{Q_{37}} - ({Q_{14}}+{Q_{24}})\cos {\Omega _2}+({Q_{18}} - {Q_{32}} - {Q_{34}})(\cos {\Omega _2} - 1) - ({Q_{29}}+{Q_{33}}+{Q_{36}} \hfill \\ \,\,\,\,\,\,\,\,\, - {Q_{38}})\sin {\Omega _2} - {Q_{23}}\sin \Omega _{2}^{2}]\cos 2\zeta +[({Q_{20}}+{Q_{35}})\cos {\Omega _2}+{Q_{12}}\sin 2{\Omega _2}+{Q_{11}}\cos \Omega _{2}^{2} \hfill \\ \,\,\,\,\,\,\,\,\, - ({Q_{15}}+{Q_{21}})\sin {\Omega _2}]\sin 2\zeta \} , \hfill \\ {\Omega _{22}}={Q_{42}} - {Q_{43}} - {Q_{39}}\cos {\zeta ^2} - {Q_{40}}\sin {\zeta ^2}+{Q_{41}}\cos \zeta . \hfill \\ \end{gathered}$$

where the constants ${Q_\hbar },\,\,(\hbar =1,2, \ldots ,43)$ are determined in Appendix C. Substituting (31), (33), and (36) into (24), and integrating with respect to $\tau$, yields

37 $$\tilde {F}={\varepsilon ^2}{\tilde {F}_2}\tau ,\quad {\Omega _1}=\mu \tau +{\varepsilon ^2}{\Omega _{12}}\tau ,\quad {\Omega _2}=\tau - {\varepsilon ^2}{\Omega _{22}}\tau .$$

The solutions of (35) is given by

38 $$\begin{gathered} {{\tilde {p}}_2}={S_1}+{c_5}\cos {\Omega _1}+{c_6}\sin {\Omega _1}+{S_2}\cos {\Omega _2}+({S_4} - \,{S_8} - {S_{10}}+{S_{11}}+{S_{15}})\cos 3(\zeta - {\Omega _1}) \hfill \\ \,\,\,\,\,\,\,\,\,\,+[3+\cos 2(\zeta - {\Omega _1})]({S_6} - {S_7}\sin {\Omega _2}) - \,[{S_8}(4\cos 2\zeta - 2) - {S_{14}}]\cos (\zeta - {\Omega _1}) \hfill \\ \,\,\,\,\,\,\,\,\,\, - {S_9}(1 - 2\cos 2\zeta )\sin (\zeta - {\Omega _1})+\,({S_5}+{S_{12}} - {S_3}\cos {\Omega _2})\cos \zeta +{S_{16}}\sin (\zeta - {\Omega _1}) \hfill \\ \,\,\,\,\,\,\,\,\,\,+2({S_4} - {S_{10}}+{S_{11}})(2\cos 2\zeta - 1)[\cos (\zeta - {\Omega _1}) - 2{\Omega _1}\sin (\zeta - {\Omega _1})] \hfill \\ \,\,\,\,\,\,\,\,\,\,+({S_{13}} - {S_{18}})\sin {\Omega _2}\sin 2(\zeta - {\Omega _1}) - {S_{17}}\sin 2(\zeta - {\Omega _1}), \hfill \\ \end{gathered}$$

39 $$\begin{gathered} {{\tilde {\alpha }}_2}={S_{19}}+3{S_{25}}+({c_7}+{S_{20}}+2{S_{30}})\cos {\Omega _2}+{c_8}\sin {\Omega _2}+6{S_{24}}\cos {\Omega _2} - {S_{36}}\sin \zeta \sin {\Omega _1} \hfill \\ \,\,\,\,\,\,\,\,\,\,+({S_{20}}+4{S_{24}}){\Omega _2}\sin {\Omega _2}+{S_{24}}\cos 3{\Omega _2} - {S_{25}}\cos 2{\Omega _2}+{S_{28}}\sin \Omega _{2}^{2}+{S_{29}}\sin 2{\Omega _2}\, \hfill \\ \,\,\,\,\,\,\,\,\,\, - ({S_{26}} - {S_{37}})\cos 2{\Omega _2}\cos (\zeta - {\Omega _1})+({S_{21}}+{S_{30}})\cos {\Omega _2}\cos 2(\zeta - {\Omega _1}) \hfill \\ \,\,\,\,\,\,\,\,\,\,+({S_{34}}+{S_{35}})\sin {\Omega _2}\cos (\zeta - {\Omega _1}) - 2({S_{22}}+{S_{32}}){\Omega _2}\cos {\Omega _2}\sin 2(\zeta - {\Omega _1}) \hfill \\ \,\,\,\,\,\,\,\,\,\, - 2({S_{34}}+{S_{35}}){\Omega _2}\cos {\Omega _2}\cos (\zeta - {\Omega _1})+({S_{22}}+{S_{32}})\sin {\Omega _2}\sin 2(\zeta - {\Omega _1}) \hfill \\ \,\,\,\,\,\,\,\,\,\,+2{S_{21}}{\Omega _2}\sin {\Omega _2}\cos 2(\zeta - {\Omega _1}) - {S_{27}}\sin 2{\Omega _2}\sin (\zeta - {\Omega _1}) - 2{S_{31}}\cos \zeta \cos (\zeta - {\Omega _1}) \hfill \\ \,\,\,\,\,\,\,\,\,\, - {S_{23}}\sin 2{\Omega _2}\sin 2(\zeta - {\Omega _1}) - 2{S_{33}}\cos {\Omega _2}\cos (\zeta - {\Omega _1}) - {S_{33}}{\Omega _2}\sin {\Omega _2}\cos (\zeta - {\Omega _1}) \hfill \\ \,\,\,\,\,\,\,\,\,\,+{S_{23}}{\Omega _2}{\cos ^2}{\Omega _2}\sin 2(\zeta - {\Omega _1})+{S_{31}}{\Omega _2}\sin {\Omega _2}{\cos ^2}(\zeta - {\Omega _1}) \hfill \\ \,\,\,\,\,\,\,\,\,\, - {S_{36}}\sin {\Omega _1}\sin (3\zeta - 2{\Omega _1})+[2{S_{33}}+3({S_{26}}+{S_{37}})]\cos (\zeta - {\Omega _1}). \hfill \\ \end{gathered}$$

The substitution of (34), (37), (38), and (39) into (23) yields the periodic solutions${p_2}$ and ${\alpha _2}$. Likewise, using (11) and (12) together with ${p_2}$ and ${\alpha _2}$, to write the solutions ${p_1},{q_1},{r_1},{\alpha _1},{\beta _1},$ and ${\gamma _1}$ as

40 $$\begin{aligned} p_{1} & = \tilde{F}\cos (\Omega _{1} - \zeta ) + \varepsilon (\tilde{p}_{1} + \xi + \xi _{2} \tilde{H}_{3} \cos \Omega _{2} ) + \varepsilon ^{2} (\tilde{p}_{2} + \xi _{2} \tilde{\alpha }_{1} ) + \varepsilon ^{3} \xi _{2} \tilde{\alpha }_{2} \\ & + \cdots ,q_{1} = - \tilde{F}N\sin (\zeta - \Omega _{1} ) + \varepsilon S_{{53}} + \varepsilon ^{2} \{ S_{{55}} - S_{{45}} + (S_{{38}} - S_{{54}} )\sin (2\zeta - \Omega _{1} )\sin \Omega _{1} \\ & + S_{{39}} {\mkern 1mu} {\mkern 1mu} {\mkern 1mu} {\mkern 1mu} {\mkern 1mu} {\mkern 1mu} {\mkern 1mu} {\mkern 1mu} {\mkern 1mu} + (S_{{40}} + S_{{42}} - S_{{57}} - {\mkern 1mu} S_{{59}} )(\cos \Omega _{2} - 1) - (S_{{41}} + S_{{43}} + S_{{50}} - S_{{58}} + S_{{62}} )\sin \Omega _{2} \} {\mkern 1mu} {\mkern 1mu} {\mkern 1mu} \\ {\mkern 1mu} & \times \sin (\zeta - \Omega _{1} ) + \varepsilon ^{2} \{ S_{{55}} - S_{{45}} - S_{{51}} + (S_{{46}} - S_{{60}} )\cos \zeta + S_{{44}} \sin (2\zeta - \Omega _{1} )\sin \Omega _{1} {\mkern 1mu} {\mkern 1mu} {\mkern 1mu} {\mkern 1mu} {\mkern 1mu} {\mkern 1mu} {\mkern 1mu} {\mkern 1mu} \\ & + [(S_{{47}} + S_{{51}} ) - (S_{{48}} + S_{{60}} )\cos (\zeta - \Omega _{1} )]\cos \Omega _{2} + (S_{{61}} - S_{{49}} - S_{{52}} - S_{{56}} )\sin \Omega _{2} \} , \\ & r_{1} = 1 + \varepsilon ^{2} \{ S_{{63}} \sin (2\zeta - \Omega _{1} )\sin \Omega _{1} + S_{{64}} (1 - \cos \Omega _{2} ) + S_{{65}} \sin \Omega _{2} \} + \cdots , \\ & \alpha _{1} = \tilde{H}_{3} \cos \Omega _{2} + \varepsilon [\tilde{\alpha }_{1} + \Lambda _{1} \tilde{F}\cos (\Omega _{1} - \zeta )] + \varepsilon ^{2} (\tilde{\alpha }_{2} + \Lambda _{1} \tilde{p}_{1} ) + \varepsilon ^{3} \Lambda _{1} \tilde{p}_{2} + \cdots , \\ & \beta _{1} = - \tilde{H}_{3} \sin \Omega _{2} + \varepsilon \tilde{F}\Lambda _{2} \sin (\zeta - \Omega _{1} ) + \varepsilon ^{2} \{ S_{{66}} + [{\mkern 1mu} S_{{72}} + S_{{67}} (1 - \cos \Omega _{2} ) + S_{{69}} \sin \Omega _{2} {\mkern 1mu} {\mkern 1mu} {\mkern 1mu} {\mkern 1mu} {\mkern 1mu} {\mkern 1mu} {\mkern 1mu} {\mkern 1mu} {\mkern 1mu} {\mkern 1mu} {\mkern 1mu} \\ & + S_{{68}} [\sin \zeta ^{2} + \sin (\zeta - \Omega _{1} )^{2} {\mkern 1mu} ] - S_{{70}} \sin (2\zeta - \Omega _{1} )\sin \Omega _{1} ]\sin \Omega _{2} \\ & - S_{{71}} [2\cos \zeta {\mkern 1mu} {\mkern 1mu} {\mkern 1mu} {\mkern 1mu} {\mkern 1mu} {\mkern 1mu} \times \sin (\zeta - \Omega _{1} ) - \cos \Omega _{2} \sin 2(\zeta - \Omega _{1} )]\} , \\ & \gamma _{1} = 1 - \varepsilon \{ S_{{73}} \sin \Omega _{2} \sin (\zeta - \Omega _{1} ) - S_{{74}} [\cos \zeta - \cos \Omega _{2} \cos (\zeta - \Omega _{1} )]\} + \varepsilon ^{2} S_{{75}} . \\ \end{aligned}$$

where ${c_f}\,(f=1,2, \ldots ,8)$ are arbitrary constants of the general solutions of Eqs. (30) and (35). These solutions demonstrate the significance of the period correction $\Xi (\varepsilon )$ as

41 $$\Xi (\varepsilon )= - \varepsilon \pi n\{ {S_{76}} - {S_{77}}\} .$$

where the constants ${S_z},\,\,(z=1,2, \ldots ,77)$ are determined in Appendix D.

It is imperative to remember that the attained solutions (40) and (43) are in excellent agreement with those in^21,22^ for the scenario of the movement of an uncharged RB in a UGF without the GT’s effectiveness. These outcomes are therefore regarded as a generalization of these investigations.

It is important to note that these solutions are given in terms of trigonometric functions. Then we predict that they have periodic behaviors, which are what we aimed for, and this will also be confirmed through their graphical representations for different values of the parameters affecting the motion, as discussed below. Moreover, the importance of having periodic solutions will be highlighted, along with the implications for various spacecraft applications. These solutions are valid for any value of the body’s frequency, and they are completely free from any singular points. The main reason is due to the use of Amer’s frequency^21^, which directly depends on the third component of the GT.

## Euler’s angles

This section focuses on getting explicit approximations of Euler angles $\theta ,\psi ,$ and $\phi$, as a power series of $\varepsilon$, in accordance with the aforementioned solutions in order to illustrate the RB’s motion at every specified instant. Therefore, we are going to recall the mathematical formula of these angles^1–3^

42 $$\theta ={\cos ^{ - 1}}\gamma ,\quad \,\,\,\,{\phi _0}={\tan ^{ - 1}}\frac{{{\alpha _0}}}{{{\beta _0}}},\,\,\,\,\,\,\,\,\,\,\,\,\frac{{d\psi }}{{dt}}=\frac{{p\alpha +q\gamma }}{{1 - {\gamma ^2}}},\,\,\,\,\,\,\,\,\,\,\,\,\frac{{d\phi }}{{dt}}=r - \frac{{d\psi }}{{dt}}\cos \theta ,$$

Transforming these angles into the scaled nondimensional form as

43 $$\theta ={r_0}\,{\theta _1},\,\,\,\,\,\,\,\,\,\,\,\,\,\,\,\,\,\,\,\,\,\,\,\,\phi ={r_0}\,{\phi _1},\,\,\,\,\,\,\,\,\,\,\,\,\,\,\,\,\,\,\,\,\,\,\,\,\,\,\,\psi ={r_0}\,{\psi _1}.$$

Using (4) and the last equation in (40), the expression of the angle of rotation $\theta$ is presented in the form

44 $$\begin{gathered} {\theta _1}=r_{0}^{{ - 1}}{\cos ^{ - 1}}\{ {\gamma _0}[1 - \varepsilon \{ {S_{73}}\sin {\Omega _2}\sin (\zeta - {\Omega _1}) - {S_{74}}[\cos \zeta - \cos {\Omega _2}\cos (\zeta - {\Omega _1})]\} \hfill \\ \quad +{\varepsilon ^2}{S_{75}}]\} . \hfill \\ \end{gathered}$$

Transforming the differentiation for the angles $\psi$ and $\phi$ with respect to $\tau$ implies

45 $${\dot {\psi }_1}=\varepsilon {r_0}{\gamma _0}\frac{{{p_1}{\alpha _1}+{q_1}{\beta _1}}}{{1 - \gamma _{0}^{2}\gamma _{1}^{2}}},\quad {\dot {\phi }_1}={r_0}{r_1} - {\gamma _0}{\gamma _1}{\dot {\psi }_1}.$$

Applying the KBM approach, the expressions of ${\psi _1}$ and ${\phi _1}$ can be expressed in terms of $\varepsilon$ as

46 $${\psi _1}=\sum\limits_{{n=1}}^{d} {{\varepsilon ^n}} {\psi _{1n}}(\tilde {F},{\Omega _1})+O({\varepsilon ^{d+1}}),\quad {\phi _1}=\sum\limits_{{n=1}}^{d} {{\varepsilon ^n}} {\phi _{1n}}(\tilde {F},{\Omega _2})+O({\varepsilon ^{d+1}}).$$

Estimation of the functions ${\dot {\psi }_1}$ and ${\dot {\phi }_1}$, produces

47 $${\dot {\psi }_1}=\frac{{d\tilde {F}}}{{d\tau }}\frac{{\partial {\psi _1}}}{{\partial \tilde {F}}}+\frac{{d{\Omega _1}}}{{d\tau }}\frac{{\partial {\psi _1}}}{{\partial {\Omega _1}}},{\text{ }}\quad \quad \quad \quad \,\,\,\,\,\,\,\,\,\,\,{\text{ }}{\dot {\phi }_1}=\frac{{d\tilde {F}}}{{d\tau }}\frac{{\partial {\phi _1}}}{{\partial \tilde {F}}}+\frac{{d{\Omega _2}}}{{d\tau }}\frac{{\partial {\phi _1}}}{{\partial {\Omega _2}}},$$

Making use of (11), (12), (38), (39), and (40) into (45), and getting rid of terms that create secular ones, yields

48 $$\begin{gathered} {\psi _{11}}={V_1}[N\cos (\zeta - {\Omega _1})\sin {\Omega _2} - \cos {\Omega _2}\sin (\zeta - {\Omega _1})], \hfill \\ {\psi _{12}}=2{V_4}{\Omega _2} - {V_7}+{V_2}{\Omega _1}\cos {\Omega _2}+{V_4}\sin 2(\zeta - {\Omega _2}) - ({V_8}+{V_{16}})\cos 2{\Omega _2} - \,[{V_3}{\Omega _1} \hfill \\ \,\,\,\,\,\,\,\,\,\,\,\,\,\,+{V_5}\sin {\Omega _2}+{V_6}\cos {\Omega _2}]\sin {\Omega _2} - ({V_9}+{V_{17}})\cos 2(\zeta - {\Omega _1})\sin 2{\Omega _2}+({V_{10}}+{V_{18}}) \hfill \\ \,\,\,\,\,\,\,\,\,\,\,\,\,\, \times \cos \zeta \cos (\zeta - {\Omega _1})\sin {\Omega _2} - ({V_{12}}+{V_{19}})\cos \zeta \cos {\Omega _2}\sin (\zeta - {\Omega _1})+({V_{11}}+{V_{20}}) \hfill \\ \,\,\,\,\,\,\,\,\,\,\,\,\,\, \times \sin 2(\zeta - {\Omega _1})+({V_{13}}+{V_{21}})\cos 2{\Omega _2}\sin 2(\zeta - {\Omega _1}) - \,({V_{14}}+{V_{22}})\sin 2{\Omega _2} \hfill \\ \,\,\,\,\,\,\,\,\,\,\,\,\,\, - \,({V_{15}}+{V_{23}})\sin 2\Omega _{2}^{2}, \hfill \\ \end{gathered}$$

and

49 $$\begin{gathered} {\phi _{11}}={V_{24}}[\cos (\zeta - {\Omega _1})\sin {\Omega _2} - N\cos {\Omega _2}\sin (\zeta - {\Omega _1})], \hfill \\ {\phi _{12}}=({V_{26}}+{V_{27}} - {V_{48}}){\Omega _2} - ({V_{26}}+{V_{27}} - {V_{28}})\sin {\Omega _2} - ({V_{25}}+{V_{29}} - {V_{30}})\cos {\Omega _2} \hfill \\ \,\,\,\,\,\,\,\,\, - {V_{31}}{\Omega _2}\sin {\Omega _2}\sin (2\zeta - {\Omega _1}) - {V_{43}}{\Omega _2}[\sin 2\zeta \sin 2{\Omega _1}+\cos 2\zeta \cos 2{\Omega _1}] \hfill \\ \,\,\,\,\,\,\,\,\, - 2({V_{32}} - {V_{34}}+{V_{36}}+{V_{37}}+{V_{38}}+{V_{39}}){\Omega _2}+2{\cos ^2}\zeta [{V_{42}}\sin {\Omega _1}\cos {\Omega _2} \hfill \\ \,\,\,\,\,\,\,\,\,+{V_{41}}\cos {\Omega _1}\sin {\Omega _2}] - ({V_{32}} - {V_{34}}+{V_{36}} - {V_{37}}+{V_{38}} - {V_{40}}+{V_{48}})\sin 2{\Omega _2} \hfill \\ \,\,\,\,\,\,\,\,\,+\sin 2\zeta [{V_{41}}\sin {\Omega _1}\sin {\Omega _2} - {V_{42}}\cos {\Omega _1}\cos {\Omega _2}]+{V_{47}}\cos 2{\Omega _2} \hfill \\ \,\,\,\,\,\,\,\,\, - \sin 2\zeta [{V_{44}}\sin 2{\Omega _1}\sin 2{\Omega _2} - {V_{46}}\cos 2{\Omega _1}\cos 2{\Omega _2}]+{V_{33}}{\Omega _2}\sin {(\zeta - {\Omega _1})^2} \hfill \\ \,\,\,\,\,\,\,\,\, - \cos 2\zeta [{V_{45}}\sin 2{\Omega _1}\cos 2{\Omega _2}+{V_{44}}\cos 2{\Omega _1}\sin 2{\Omega _2}]. \hfill \\ \end{gathered}$$

where the constants ${V_w},\,\,(w=1,2, \ldots ,48)$ are determined in Appendix E.

Based on the above expressions of Euler angles, one can say that these expressions provide a way to represent the RB’s orientation in 3D space, facilitating the calculation of necessary maneuvers to achieve desired positions or orientations. Accurate expressions are essential for tasks such as RB stabilization, docking procedures, and navigation through space.

## Outcome discussion

This section’s purpose is to analyze and outline the demonstrated simulated plots of the obtained results (40) and (46) according to the influence of the RB’s parameters. To accomplish this task, we have used Wolfram Mathematica 13.2 and considered the following numerical data for some of the independent parameters.

$$\begin{gathered} D = (90,80,65){\text{kg m}}^{2} ,\,\,\,x_{0} = 0.2{\text{m}},\,\,y_{0} = 0.5{\text{m}},\,\,z_{0} = 0.7{\text{m}},\,\,\,m = 150\,{\text{kg}},\,\,\,\Gamma = 0.1\text{s} ^{{ - 2}} , \hfill \\ \lambda _{3} ( = 1000,1100,1200){\text{kg m}}^{2} {\text{ s}}^{{ - 1}} ,\,\,\,\,\delta ( = 100,150,200){\text{C}},\,\,\,\,\,\,g = 9.8{\text{m s}}^{{ - 2}} ,\,\,\,\,\,c_{f} = 0,\, \hfill \\ r_{0} = 500\,{\text{rad s}}^{{ - 1}} ,\,\,\,\gamma _{0} = {\text{0}}{\text{.001,}}\,\,\,H_{1} = 0.003,\,\,\,H_{2} = 0.008,\,\,\,\,\psi _{0} = 0,\,\,\,\,\phi _{0} = 0,\,\,\,\,\tau \in [0,40]\,{\text{s}}{\text{.}} \hfill \\ \end{gathered}$$

**Fig. 2:**
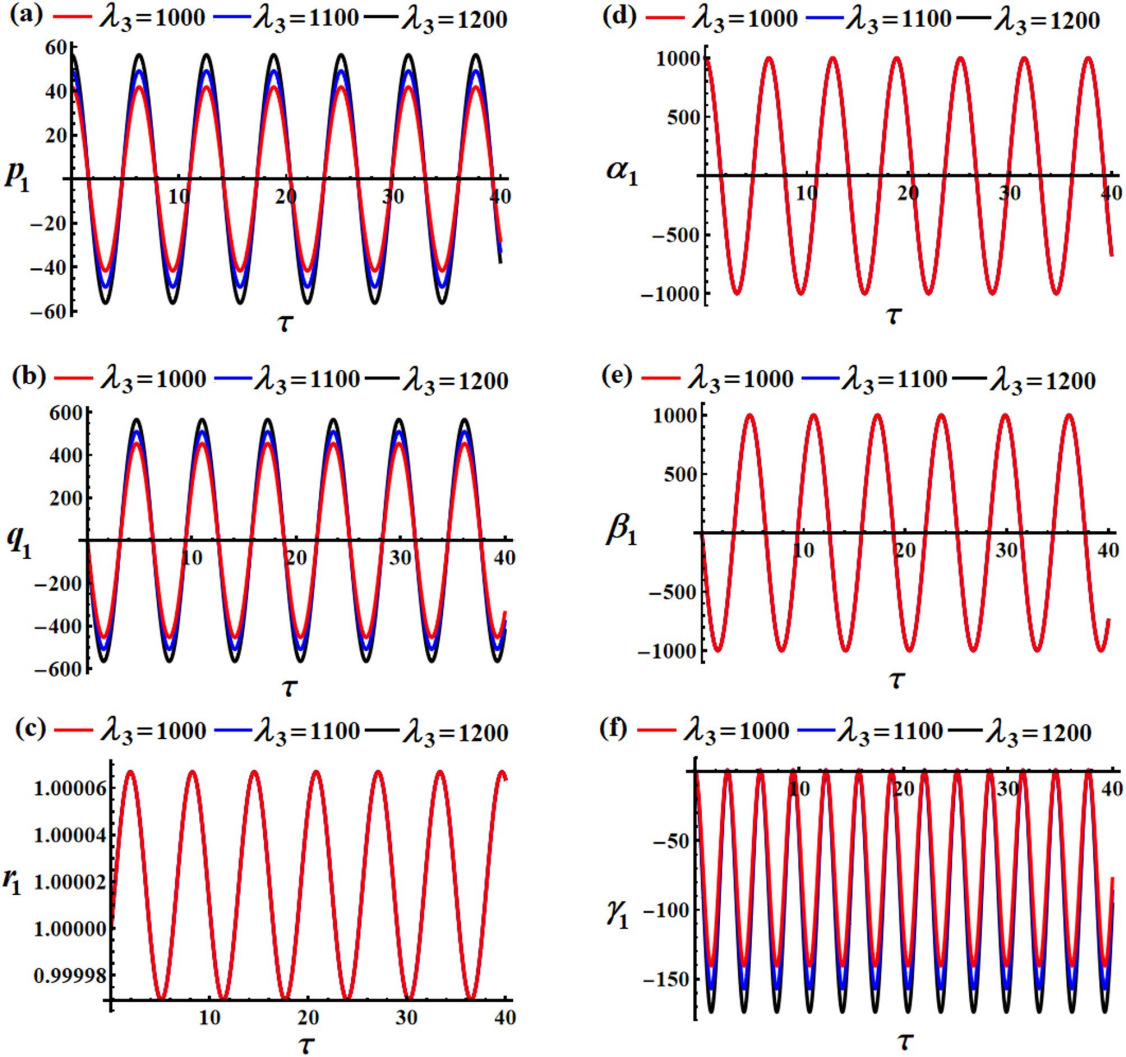
The influence of ${\lambda _3}(=1000,1100,1200){\text{kg}}{\text{.}}{{\text{m}}^2}.{{\text{s}}^{ - 1}}$on (**a**) ${p_1}$, (**b**) ${q_1}$, (**c**) ${r_1}$, (**d**) ${\alpha _1}$, (**e**) ${\beta _1}$, and (**f**) ${\gamma _1}$.

**Fig. 3:**
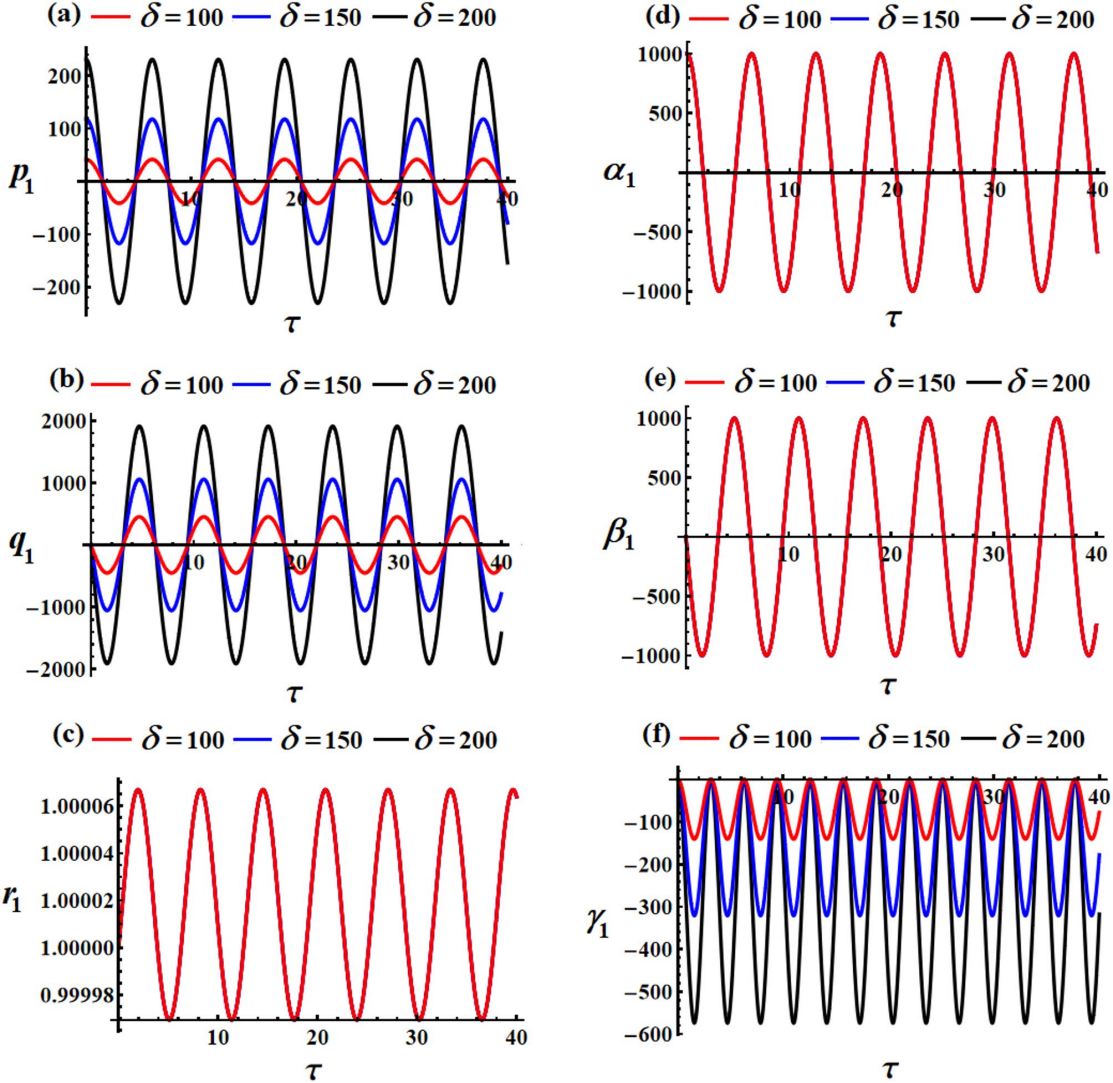
The impact of $\delta (=100,150,200){\text{C}}$on (**a**) ${p_1}$, (**b**) ${q_1}$, (**c**) ${r_1}$, (**d**) ${\alpha _1}$, (**e**) ${\beta _1}$, and (**f**) ${\gamma _1}$.

Curves of Figs. 2 and 3 show the variation of the body’s time histories, when $\delta (=100,150,200){\text{C}}$ and ${\lambda _3}(=1000,1100,1200){\text{kg}}{\text{.}}{{\text{m}}^2}.{{\text{s}}^{ - 1}}$. It must be mentioned that these curves have periodic behaviors, as predicted before, throughout the whole period. The rationale stems from the mathematical expressions of the obtained outcomes (40) are provided using trigonometric functions. The variation of the values of the GT ${\lambda _3}$ has a positive impact on the solutions ${p_1}$ and ${q_1}$, and ${\gamma _1}$, as drawn in Fig. 2a,b,f. The inspection of the waves describing the solution ${p_1}$ reveals that they have a form of standing waves. These waves’ amplitudes rise in proportion to ${\lambda _3}$ while the value of ${p_1}$ rises as well. In addition, the wave’s number remains steady without change. Separated waves are drawn in the graph describing the solution ${q_1}$ increases, where their amplitude increases with the raised values of ${\lambda _3}$, While the value of ${\gamma _1}$ decreases with the increase of the GT. In contrast, the other solutions don’t alter as these GT values do because they are not depending on ${\lambda _3}$ explicitly or having a higher power larger than ${\varepsilon ^3}$, see Fig. 2c,d,e.

The system appears to be exhibiting gyroscopic precession where:

- The GT ${\lambda _3}$ acts as a stabilizing influence.
- Increasing ${\lambda _3}$ increases the precession frequency without significantly affecting the motion amplitude.
- The motion is quasi-periodic with dominant oscillations in two directions and smaller perturbations in the third.
- This behavior is typical of spinning rigid bodies with gyroscopic effects, such as satellites with momentum wheels or spinning tops.

A closer examination of the parts of Fig. 3 shows that the beneficial result that followed changing $\delta$-values is observed in the curves that describe the solutions ${p_1},{q_1},$ and ${\gamma _1}$, as graphed in parts (a), (b), and (f). As mentioned before, periodic curves have been plotted, as predicted before, in the forms of standing waves, see Fig. 3a, where the resultant waves’ amplitude increases in proportion to $\delta$-values as ${p_1}$ increases as well. The change of the solution ${q_1}$ is explored in Fig. 3b. Whereas the variation of the waves describing solution ${\gamma _1}$ becomes evident when $\delta$ have various values, wherein these waves’ amplitudes rise in proportion to elevated values of $\delta$ while its value decreases, as noted in Fig. 3f. As for the remaining solutions ${r_1},{\alpha _1},$ and ${\beta _1}$, we find that their waves do not suffer from any change that occurs with the $\delta$ values, as portrayed in Fig. 3c–e. The system shows how MFs can be used for attitude control, where increasing field strength provides stronger control authority but can also induce larger oscillations if not properly damped. The MF acts as an energy pump, driving larger oscillations without changing the natural frequency.

**Fig. 4:**
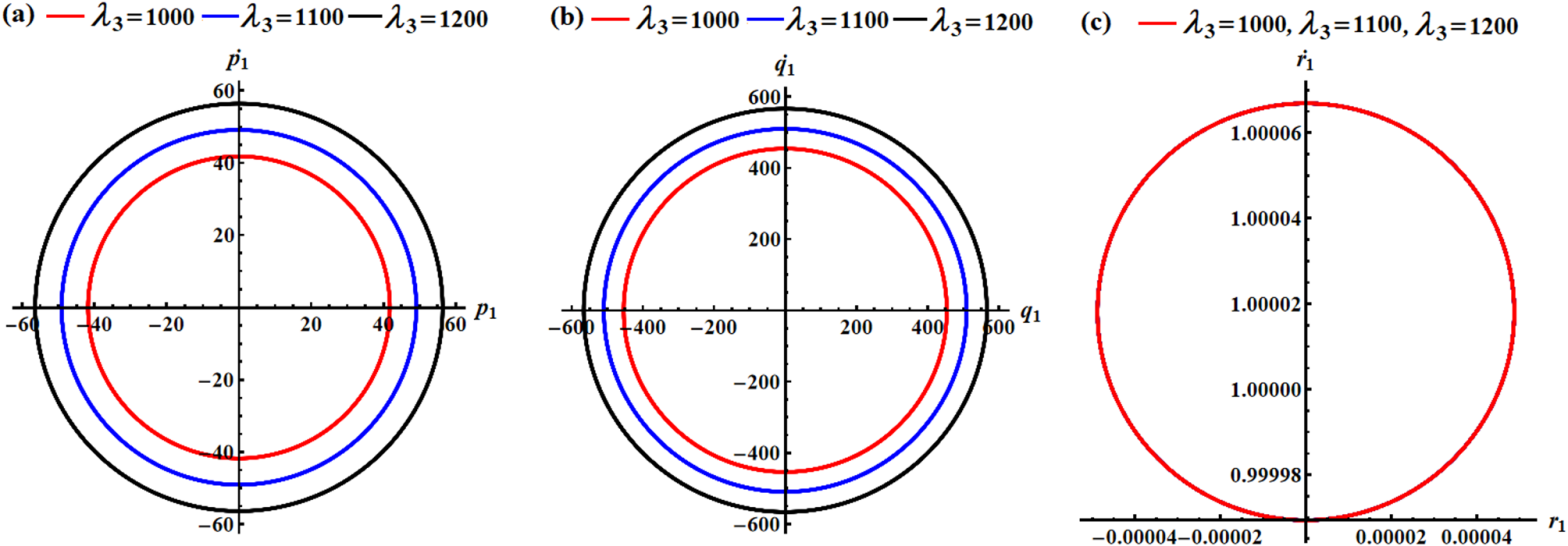
The phase simulations of the graphed AS of parts (a), (b), and (c) in Fig. 2.

**Fig. 5:**
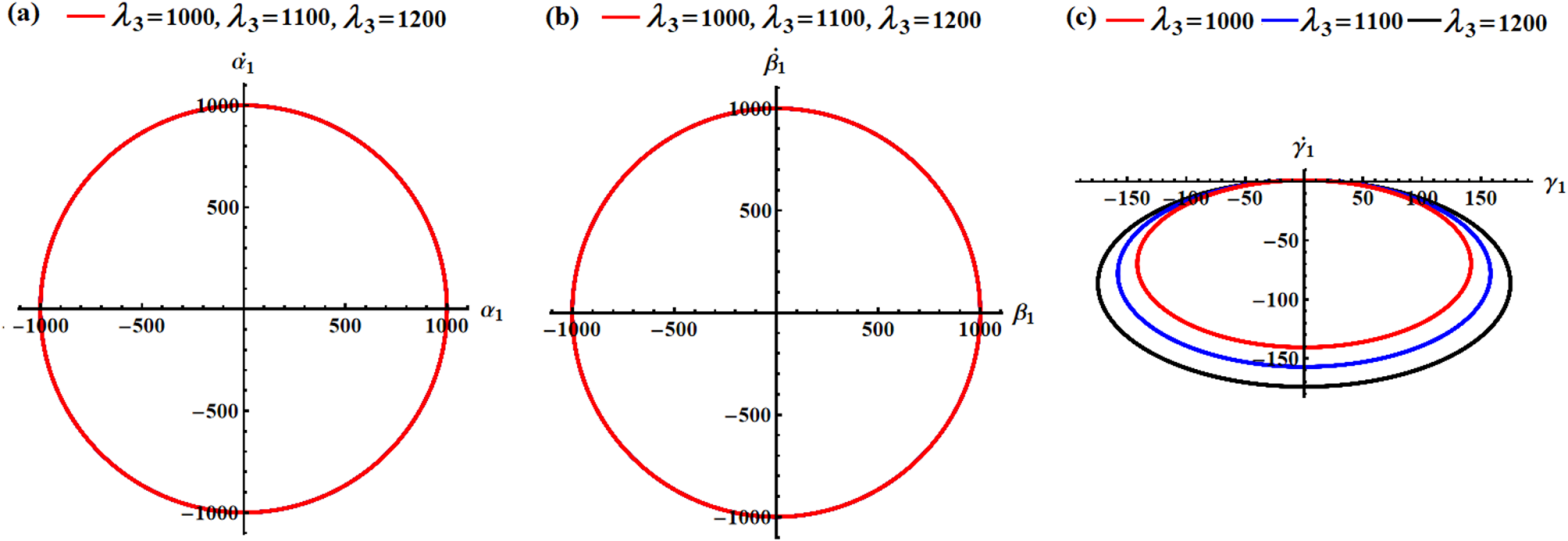
The phase portraits of the graphed AS of parts (d), (e), and (f) in Fig. 2.

**Fig. 6:**
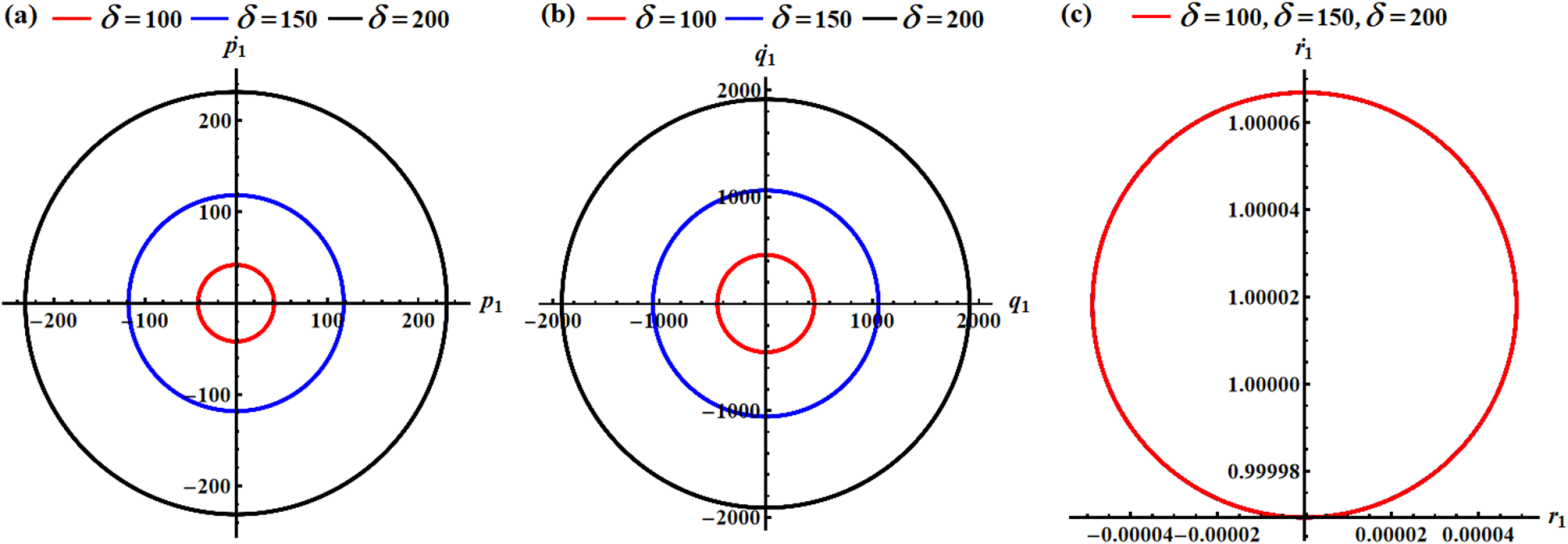
The phase diagrams of the graphed AS of parts (a), (b), and (c) in Fig. 3.

**Fig. 7:**
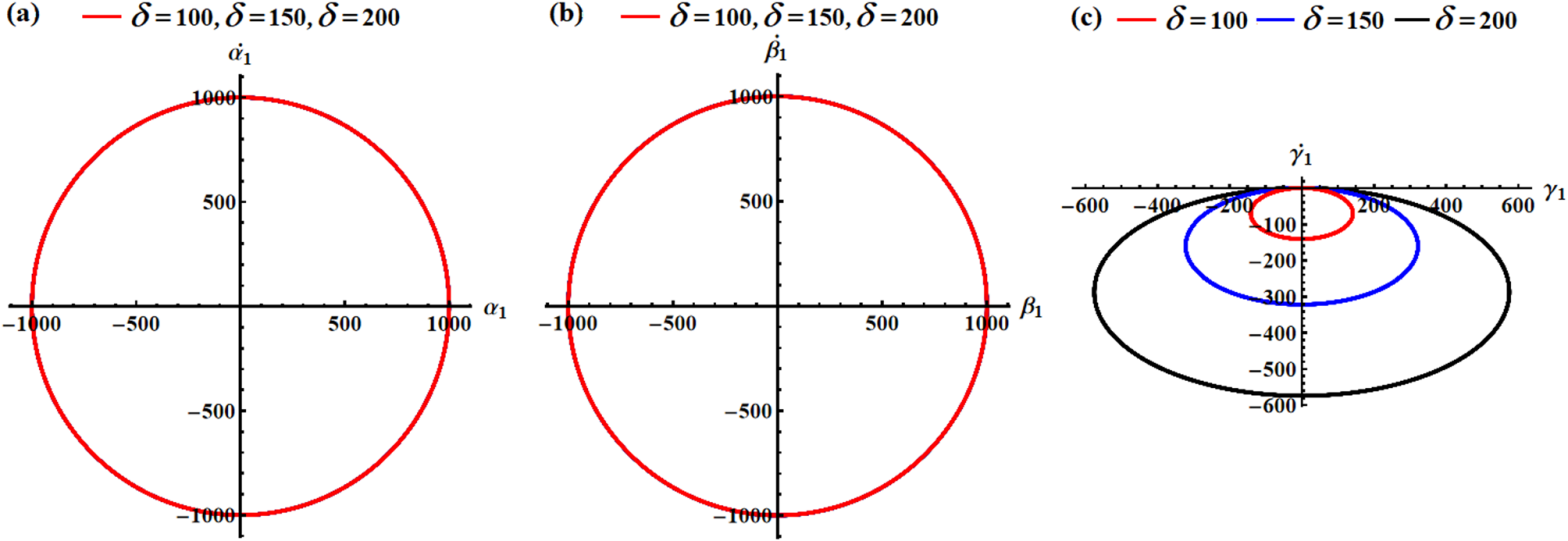
The phase diagrams of the graphed AS of parts (d), (e), and (f) in Fig. 3.

In light of the aforementioned figures, one can draw the diagrams of phase-plane for the AS that are plotted in Figs. 2 and 3. To accomplish this aim, the curves of Figs. 4, 5, 6 and 7 are graphed in the plane of the attained solutions and their first derivatives. The inspection of the parts of these figures demonstrated that we have obtained a closed symmetric curve for each solution whether with the change of ${\lambda _3}$-values or with $\delta$-values. These phase curves assert that the solutions have stable manners, and the variation of these curves goes back to the variation of their solutions. Increasing ${\lambda _3}$-values consistently reduces oscillation amplitudes in all rotational components, demonstrating the stabilizing effect of the GT. The near-constant ${r_1}$ resulting that the RB is in a spin-stabilized regime where rotation about one axis dominates. The different scales and patterns across phase planes reveal the RB has different moments of inertia, creating asymmetric dynamic responses which consists with the simulation values. Higher $\delta$ values create stronger MF, reducing oscillation amplitudes similar to a magnetic bearing system. All trajectories remain closed orbits, indicating the MF provides passive stabilization without causing instability. The concentric nature shows that the MF primarily affects amplitude, not frequency of oscillations.

In conclusion, the GT acts as a dynamic stabilizer, reducing oscillation amplitudes across all motion components through gyroscopic stiffening. Higher ${\lambda _3}$ values create tighter, more controlled orbits while maintaining the fundamental motion patterns. The MF parameter functions as a selective damper, primarily constraining transverse motions $({p_1},{q_1})$and vertical orientation ${\gamma _1}$ while leaving spin motion ${r_1}$ virtually unaffected. Lower $\delta$ values allow larger amplitude oscillations, demonstrating the MF’s role as a restoring force.

**Fig. 8:**
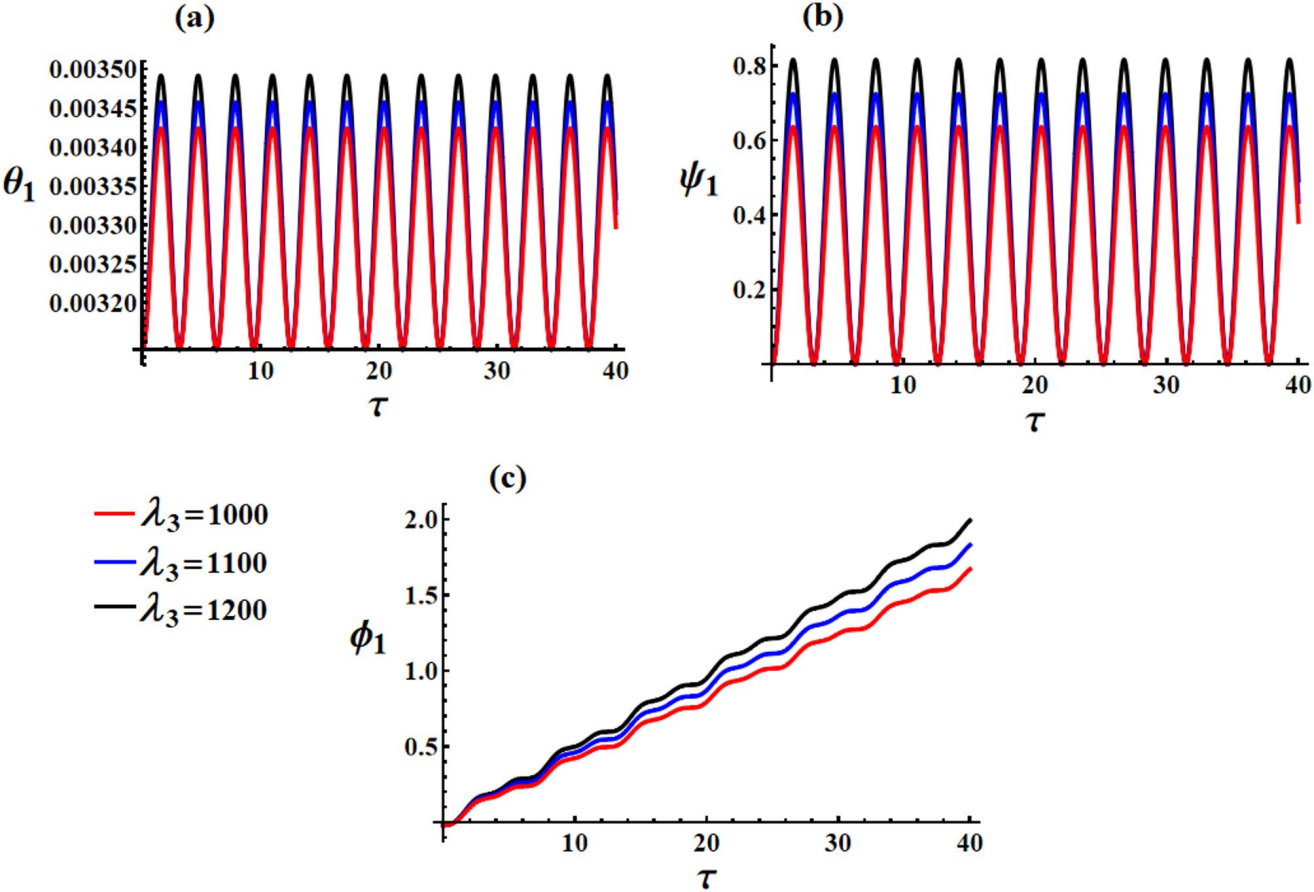
The impact of ${\lambda _3}(=1000,1100,1200){\text{kg}}{\text{.}}{{\text{m}}^2}.{{\text{s}}^{ - 1}}$on Euler angles at $\tau \in [0,40]{\text{s}}$.

**Fig. 9:**
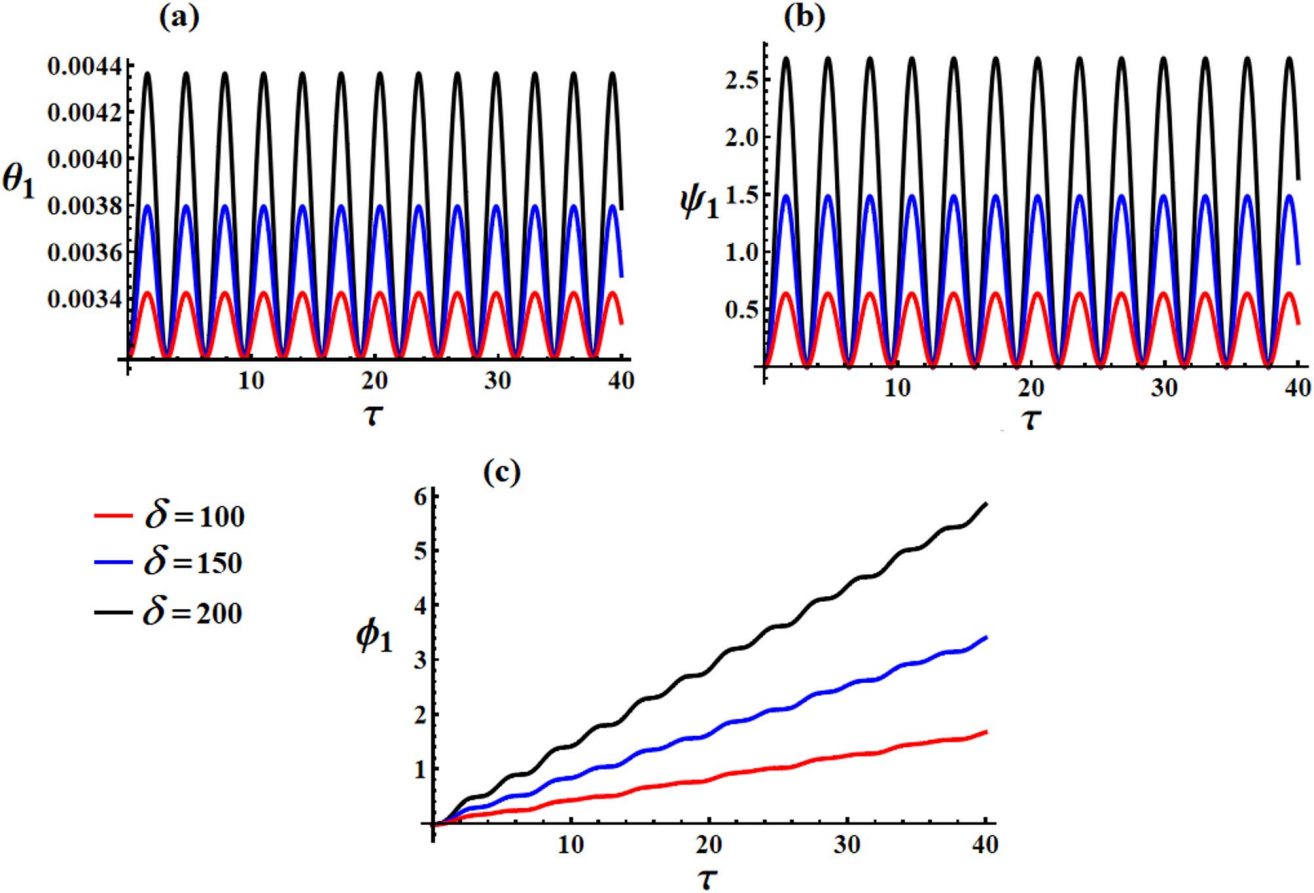
The influence of $\delta (=100,150,200){\text{C}}$ on the angles: (**a**) ${\theta _1}$, (**b**) ${\psi _1}$, and (**c**) ${\phi _1}$ at $\tau \in [0,40]{\text{s}}$.

Parts of Figs. 8 and 9 provide us the temporal variation of the obtained scaled Euler angels (44) and (46) in light of the previous data and various values of ${\lambda _3}(=(1000,1100,12000){\text{kg}}{\text{.}}{{\text{m}}^2}.{{\text{s}}^{ - 1}}$and $\delta (=100,150,200){\text{C}}$, respectively. It is significant to state that the behavior of the scaled nutation angle ${\theta _1}$ has the form of other standing-increasing waves, as drawn in Fig. 8a. We find that by increasing the values of ${\lambda _3}$, the waves’ amplitude increases, and the value of ${\theta _1}$ also increases. On the other side, the waves describing scaled precession angle ${\psi _1}$ behave the forms of standing periodic curves, as seen in Fig. 8b, in which the waves’ amplitude increases and the ${\psi _1}$ value increases with the increase of ${\lambda _3}$. The proper-rotation angle ${\phi _1}$ increases gradually during the investigated period, as observed in Fig. 8c. Higher ${\lambda _3}$ systematically reduces oscillation amplitudes in all angles. The GT increases all characteristic frequencies. The system exhibits textbook spinning top dynamics with precession, nutation, and stabilization effects clearly visible.

The influence of various values of $\delta (=100,150,200){\text{C}}$ on the behavior of scaled Euler angles ${\theta _1},{\psi _1},$ and ${\phi _1}$ is presented in Fig. 8a–c, respectively. It is obvious that this influence is obvious, as the increase in $\delta$-values implies an increase in all the angle values. The behavior of ${\theta _1}$ and ${\psi _1}$ has the forms of standing periodic waves where its amplitudes increase gradually through the examined interval, respectively. Whereas ${\phi _1}$ grows linearly with the same examined interval. The explanation stems from the mathematical constructions of these angles, as in equations (46). Unlike ${\lambda _3}$ which affects both amplitude and frequency, $\delta$ only controls amplitude while preserving natural frequencies. The MF acts as a position-dependent restoring force-stronger field creates tighter confinement around equilibrium positions without changing the system’s natural dynamics.

The effect of various NFF values on the angular velocities of the RB appears to be very small because all terms containing it in the obtained solutions correspond to the coefficient of third or higher power of $\varepsilon$.

## Conclusion

The 3D rotatory motion of an asymmetric RB about one stationary point has been examined as a generic case, in response to GT about the body’s principal axis, NFF, and MF. It has been assumed that the RB has initially a high-value angular velocity about the third main-inertia axis. The novel AS of the regulating EOMs has been obtained using the KBM approach. Unlike the earlier research^21^, these approaches are suitable for any value of the RB frequency and are devoid of singularities. Therefore, the obtained results generalize these works in addition to the results in^22^ for the cases of an uncharged body in a UGF and the absence of the GT. These solutions have been plotted and discussed in light of the acted torque and forces which has given important feedback on how to control the motion or maintain its stability. The discussion of the motion’s interpretation has been presented according to Euler angles, which describe the body’s orientation at each instant given the graphical depiction of these angles. The expressions achieved for angular velocities and Euler angles are considered crucial for spacecraft motion and application due to their essential role in attitude determination and control. These expressions describe how the spacecraft’s orientation changes over time, enabling precise adjustments to its trajectory and providing a way to represent the spacecraft’s orientation in 3D space, thereby aiding in calculating necessary maneuvers to reach desired positions or orientations. Additionally, they play a vital role in satellite stabilization, docking procedures, and navigation through space and enable spacecraft to maintain proper alignment for communication, scientific observations, and mission-critical operations. Essentially, mastering these mathematical tools allows for precise control and efficient functioning of spacecraft in the vast and challenging environment of space.

## Supplementary Information

Below is the link to the electronic supplementary material.

Supplementary Material 1

## Data Availability

The datasets used and/or analysed during the current study available from the corresponding author on reasonable request.

## Abbreviations

RB: Rigid body
GT: Gyrostatic torque
EOMs: Equations of motion
NFF: Newtonian force field
KBM: Krylov–Bogoliubov–Mitropolski
MF: Magnetic field
AS: Analytical solution
3D, 2D, 1D: Three dimensions, two dimensions, one dimension
DEs: Differential equations
PMSP: Poincaré method of small parameter
UGF: Uniform gravitational field
CBFTs: Constant body fixed torques
$D=({D_1},{D_2},{D_3})$: Uniform gravitational field
$\underline {{\hat {\kappa }}} =(\alpha ,\;\beta ,\gamma )$: The unit vector oriented along ${z_1}$-axis
$$\underset{\raise0.3em\hbox{$\smash{\scriptscriptstyle-}$}}{\sigma } =(p,q,r)$$: The RB’s angular velocity vector
$$(\psi ,\phi ,\theta )$$: Euler angles
$$\underset{\raise0.3em\hbox{$\smash{\scriptscriptstyle-}$}}{r} =({x_0},{y_0},{z_0})$$: The position of the RB’s center of mass
$\Gamma ={{3g} \mathord{\left/ {\vphantom {{3g} J }} \right. \kern-0pt} J }$: The attraction coefficient of the NFF
*g*: The gravitational acceleration
$J$: The distance between *O* and $O^{\prime}$
*O*: The fixed point
$O^{\prime}$: The attraction center of the NFF
$O{x_1}{y_1}{z_1}$: The fixed frame
$O{x_1}{y_1}{z_1}$: The rotational frame
$$\underset{\raise0.3em\hbox{$\smash{\scriptscriptstyle-}$}}{\lambda } =({\lambda _1},{\lambda _2},{\lambda _3})$$: The GT vector

## References

[1] Yehia, H. M. *Rigid Body Dynamics: A Lagrangian Approach* (Birkhäuser, Springer 2022).

[2] Chernousko, F. L., Akulenko, L. D. & Leshchenko, D. D. *Evolution of Motions of a Rigid Body about its Center of Mass* (Springer eBooks, 2017).

[3] Deriglazov, A. A. *Body as a Constrained System: Lagrangian and Hamiltonian Formalism* (Cambridge Scholars Publishing, 2024).

[4] Deriglazov, A. A. An asymmetrical body: example of analytical solution for the rotation matrix in elementary functions and Dzhanibekov effect. *Commun. Nonlinear Sci. Numer. Simul.* **138**, 108257 (2024).

[5] Deriglazov, A. A. Rotation matrix of a charged symmetrical body: One-parameter family of solutions in elementary functions. *Universe* **10** (6), 250 (2024).

[6] Gorr, G. V. An approach in studying gyrostat motion with variable gyrostatic moment. *Vestn Udmurt Univ. Ma Mekh. Kompyuternye Nauk.* **31** (1), 102–115 (2021).

[7] Zhong, X. et al. Analytical solutions and stability of periodic attitude motions of gyrostat spacecrafts in weakly elliptical orbits. *Commun. Nonlinear Sci. Numer. Simul* **141**, 108499 (2024).

[8] Leshchenko, D., Ershkov, S. & Kozachenko, T. Evolution of rotational motions of a nearly dynamically spherical rigid body with a moving mass. *Commun. Nonlinear Sci. Numer. Simul.* **133**, 107916 (2024).

[9] Akulenko, L. D. et al. The evolution of the motions of a rigid body close to the Lagrange case under the action of an unsteady torque. *J. Appl. Math. Mech.* **81** (2), 79–84 (2017).

[10] Leshchenko, D., Ershkov, S. & Kozachenko, T. Evolution of a heavy rigid body rotation under the action of unsteady restoring and perturbation torques. *Nonlinear Dyn.* **103** (2), 1517–1528 (2021).

[11] Amer, T. S. et al. A novel study on the fourth first integral for the rotatory motion of an impacted charged rigid body by external torques. *J. Low Freq. Noise Vib. Act. Control*. 10.1177/14613484251347080 (2025).

[12] Khan, Y. & Wu, Q. Homotopy perturbation transform method for nonlinear equations using he’s polynomials. *Comput. Math. Appl.* **61** (8), 1963–1967 (2010).

[13] Holmes, M. H. *Introduction To Perturbation Methods*, 2nd edn (Springer, 2013).

[14] Tikhonov, A. A. Rigid-body dynamics from the Euler equations to the attitude control of spacecraft in the works of scientists from Saint Petersburg state university, Part 1. *Vestnik Saint Petersburg Univ. Math. Mech. Astronomy. ***56 **(3), 322–340 (2023).

[15] Tikhonov, А. А. Rigid body dynamics from the Euler equations to the spacecraft attitude control in the works of scientists from Saint Petersburg state university, Part 2. *Vestnik Saint Petersburg Univ. Math. Mech. Astronomy*. **11** (2), 259–302 (2024).

[16] Galal, A. A. Free rotation of a rigid mass carrying a rotor with an internal torque. *J. Vib. Eng. Technol.* **11** (8), 3627–3637 (2022).

[17] Ershkov, S. V. Revisiting Euler–Poisson equations for a rigid body under the influence of time-dependent temperature field. *J. Appl. Comput. Mech.* **11** (2), 519–528 (2024).

[18] Ershkov, S. V. & Shamin, R. V. On metallic-type asteroid rotation moving in magnetic field (introducing magnetic second-grade YORP effect). *Acta Astronaut.* **224**, 195–201 (2024).

[19] Ershkov, S. & Christianto, V. Semi-analytical solving procedure for the dynamics of charged particle in parametrically variable magnetic field. *Eur. Phys. J. Plus*. **137** (8), 918 (2022).

[20] Deriglazov, A. A. Euler-Poisson equations of a dancing spinning top, integrability and examples of analytical solutions. *Commun. Nonlinear Sci. Numer. Simul.* **127**, 107579 (2023).

[21] Amer, T. S., Elkafly, H. F. & Galal, A. A. The 3D motion of a charged solid body using the asymptotic technique of KBM. *Alex Eng. J.* **60** (6), 5655–5673 (2021).

[22] Amer, T. S. & Abady, I. M. On the application of KBM method for the 3-D motion of asymmetric rigid body. *Nonlinear Dyn.* **89** (3), 1591–1609 (2017).

[23] Galal, A. A. et al. Studying the influence of a gyrostatic moment on the motion of a charged rigid body containing a viscous incompressible liquid. *Eur. Phys. J. Plus*. **138** (10), 959 (2023).

[24] Usubamatov, R. The correlation of gyroscope axial velocities. *JMM* **6** (1), 36–40 (2023).

[25] Amer, T. S. et al. Analyzing the dynamics of a charged rotating rigid body under constant torques. *Sci. Rep.* **14** (1), 9839 (2024). 38684724 10.1038/s41598-024-59857-z PMC11059353

[26] Amer, T. S. et al. Analyzing the spatial motion of a rigid body subjected to constant body-fixed torques and gyrostatic moment. *Sci. Rep.* **14** (1), 5390 (2024). 38443505 10.1038/s41598-024-55964-z PMC10914825

[27] Amer, T. S. et al. Modeling analysis on the influence of the gyrostatic moment on the motion of a charged rigid body subjected to constant axial torque. *J. Low Freq. Noise Vib. Act. Control*. **43** (4), 1593–1610 (2024).

[28] Zhong, X. et al. Stability analysis of resonant rotation of a gyrostat in an elliptic orbit under third-and fourth-order resonances. *Regul. Chaotic Dyn.* **28** (2), 162–190 (2023).

[29] Qi, G. & Yang, X. Modeling of a chaotic gyrostat system and mechanism analysis of dynamics using force and energy, *Complexity*. ** 2019**(1). (2019).

[30] Hu, W. et al. Dynamic analysis on an asymmetric spatial dumbbell-type model. *Adv. Space Res.* **74** (1), 348–358 (2024).

[31] Hu, J. et al. Dynamic analysis on continuous beam carrying a moving mass with variable speed. *J. Vib. Eng. Technol.* **11** (8), 3815–3825 (2022).

[32] Xu, M. et al. Symmetry-breaking dynamics of a flexible hub-beam system rotating around an eccentric axis. *Mech. Syst. Signal. Proc.* **222**, 111757 (2024).

[33] Amer, T. S. et al. The asymptotic solutions for the motion of a charged symmetric gyrostat in the irrational frequency case. *Sci. Rep.* **14** (1), 16662 (2024). 39030313 10.1038/s41598-024-66866-5 PMC11271634

[34] Leshchenko, D. & Kozachenko, T. Perturbed motions of a nearly dynamically spherical rigid body with a movable mass subject to constant body-fixed torque. *MMM* **6** (2), 18–30 (2024).

[35] Amer, T. S., Elneklawy, A. H. & El-Kafly, H. F. A novel approach to solving euler’s nonlinear equations for a 3DOF dynamical motion of a rigid body under gyrostatic and constant torques. *J. Low Freq. Noise Vib. Act. Control*. **44** (1), 111–129 (2024).

[36] Kosov, A. A. On the stability of stationary solutions of the equations of motion of the Goryachev-Sretensky gyrostat. *Mech. Solids*. **58**, 1986–1997 (2023).

[37] Kosov, A. A. & Semenov, E. I. On the integrability and stability of stationary solutions of the Goryachev–Sretensky gyrostat. *J. Appl. Ind. Math.* **17**, 557–570 (2023).

[38] Amer, T. S., Elneklawy, A. H. & El-Kafly, H. F. Analysis of euler’s equations for a symmetric rigid body subject to time-dependent gyrostatic torque. *J. Low Freq. Noise Vib. Act. Control*. **44** (2), 831–843 (2025).

[39] Amer, T. S. et al. Modeling of the Euler-Poisson equations for rigid bodies in the context of the gyrostatic influences: an innovative methodology. *Eur. J. Pure Appl. Math.* **18** (1), 5712 (2025).

[40] Amer, T. S. et al. Stability analysis of a rotating rigid body: the role of external and gyroscopic torques with energy dissipation. *J. Low Freq. Noise Vib. Act. Control*. **44 **(3), 1502-1515 (2025).

[41] Amer, T. S., Elneklawy, A. H. & El-Kafly, H. F. Dynamical motion of a spacecraft containing a slug and influenced by a gyrostatic moment and constant torques. *J. Low Freq. Noise Vib. Act. Control*. **44 **(3), 1708-1725 (2025).

[42] Hu, W., Song, M. & Deng, Z. Energy dissipation/transfer and stable attitude of Spatial on-orbit tethered system. *J. Sound Vibr*. **412**, 58–73 (2017).

[43] Amer, T. S. et al. Asymptotic solutions for the 3D motion of asymmetric charged gyrostatic satellite using Poincaré small parameter technique. *Aerosp. Sci. Technol. ***168**, Part A, 110764 (2025).

[44] Tikhonov, A. A. & Tkhai, V. N. Symmetric oscillations of charged gyrostat in weakly elliptical orbit with small inclination. *Nonlinear Dyn.* **85** (3), 1919–1927 (2016).

[45] Kalenova, V. I., Morozov, V. M. & Rak, M. G. Stabilization of regular satellite precessions using Lorentz force moments. *Cosm. Res.* **62** (1), 92–98 (2024).

[46] Akulenko, L. D. et al. Rapid rotations of a satellite with a cavity filled with viscous fluid under the action of moments of gravity and light pressure forces. *Cosm. Res.* **49** (5), 440–451 (2011).

[47] Akulenko, L. D., Leshchenko, D. D. & Rachinskaya, A. L. Evolution of the satellite fast rotation due to the gravitational torque in a dragging medium. *Mech. Solids*. **43** (2), 173–184 (2008).

[48] Sazonov, V. V. & Troitskaya, A. V. On periodic motions of a gyrostat satellite with a large inner (gyrostatic) angular momentum. *J. Appl. Math. Mech.* **81** (4), 295–304 (2017).

[49] Arkhangel’skii, I. A. Periodic solutions of quasilinear autonomous systems which have first integrals. *J. Appl. Math. Mech.* **27** (2), 551–557 (1963).

[50] Yamgoué, S. B. & Kofané, T. C. Application of the Krylov-Bogoliubov-Mitropolsky method to weakly damped strongly non-linear planar hamiltonian systems. *Int. J. Non Linear Mech.* **42**, 1240–1247 (2007).

