# Supplementary material for: Application of Krylov–Bogoliubov–Mitropolski method to asymmetric gyrostatic 3D motion in multi-fields
Source: Sci Rep. 2025 Nov 27;15:42548. doi: 10.1038/s41598-025-27177-5 (PMC12663385; doi:10.1038/s41598-025-27177-5)
Supplement: Supplementary file 1 — Supplementary Material 1 [file 41598_2025_27177_MOESM1_ESM.docx]

# Appendix A

$$L_{0}=\tilde{F}^{2}(D_{5}N^{2}-D_{4}), L_{1}=\upsilon\tilde{H}_{3}D_{4}, L_{2}= \upsilon\tilde{H}_{3}D_{5}, L_{3}= x'_{0}\tilde{H}_{3}, L_{4}= y'_{0}\tilde{H}_{3},$$

$$L_{5}=\tilde{F}\tilde{H}_{3}D_{5}N, L_{6}=\tilde{F}\tilde{H}_{3}D_{4}, L_{7}=\Lambda_{1}x'_{0}\tilde{F}, L_{8}=\tilde{F}D_{5}N\tilde{H}_{3}(\upsilon+z'_{0}),$$

$$L_{9}=\tilde{F}D_{4}\tilde{H}_{3}(\upsilon+z'_{0}), L_{10}=y'_{0}\Lambda_{2}\tilde{F}, L_{11}=\upsilon\tilde{F}\tilde{H}_{3}D_{4}\Lambda_{1}, L_{12}=\upsilon\tilde{F}\tilde{H}_{3}D_{5}\Lambda_{2},$$

$$L_{13}=\xi D_{4}\tilde{F}, L_{14}=\xi_{1}\tilde{H}_{3}D_{4}\tilde{F}, L_{15}=\frac{D_{5}y'_{0}\xi_{2}N^{2}\tilde{H}_{3}\tilde{F}^{2}}{D_{4}}, L_{16}=\tilde{H}_{3}D_{4}(\delta+\xi),$$

$$L_{17}=\tilde{H}_{3}D_{5}\delta, L_{18}=\tilde{H}_{3}D_{4}R\tilde{F}, L_{19}=\tilde{H}_{3}D_{5}NR\tilde{F}, L_{20}=D_{5}y'_{0}N,$$

$$L_{21}=\tilde{F}^{2}(D_{4}\Lambda_{1}+D_{5}\Lambda_{2}N), L_{22}=\tilde{H}_{3}^{2}(D_{4}\xi_{1}+D_{5}\xi_{2}N),$$

# Appendix B

$$M_{0}=\upsilon(\mu^{2}-D_{1}), M_{1}=\frac{\tilde{F}\delta[D_{2}^{2}(D_{3}+1)+D_{1}D_{3}D_{11}]}{D_{1}D_{2}}, M_{2}=\frac{\tilde{F}\tilde{H}_{3}x'_{0}D_{4}(2D_{11}^{2}r_{0}+1)}{D_{5}D_{11}r_{0}},$$

$$M_{3}= \frac{\tilde{F}\lambda_{3}\tilde{H}_{3}(D_{1}D_{11}-D_{2}D_{21})}{2D_{1}D_{2}r_{0}}+\chi+D_{2}D_{11}, M_{4}=\frac{D_{11}\tilde{F}^{2}N^{2}y'_{0}}{D_{4}D_{3}r_{0}}, M_{5}=\frac{\lambda_{3}(D_{5}\upsilon D_{21}+z'_{0})}{D_{5}D_{1}r_{0}},$$

$$M_{6}=\frac{\tilde{F}\tilde{H}_{3}y'_{0}}{D_{4}}, M_{7}=\tilde{F}\upsilon D_{11}\tilde{H}_{3}(D_{21}+1), M_{8}=\tilde{F}\upsilon D_{11}\tilde{H}_{3}N(1-D_{31}),$$

$$M_{9}=\tilde{F}^{2}(D_{4}-D_{5}N^{2}), M_{10}=\frac{\tilde{H}_{3}z'}{D_{4}}, M_{11}=\Lambda_{2}\tilde{F}^{2}\upsilon\tilde{H}_{3}, M_{12}=ND_{4}D_{11},$$

$$M_{13}=\frac{D_{1}\tilde{H}_{3}\delta}{D_{2}}, M_{14}=\frac{\tilde{F}\lambda_{3}\tilde{H}_{3}^{2}(D_{4}\xi_{1}+D_{5}N\zeta_{2})}{r_{0}D_{2}}, M_{15}= \frac{\tilde{F}\lambda_{3}\tilde{H}_{3}^{2}(D_{4}\Lambda_{1}+D_{5}N\Lambda_{2})}{r_{0}D_{2}},$$

$$M_{16}=\frac{x'_{0}}{D_{5}}, M_{17}=\frac{\tilde{F}^{2}N\tilde{H}_{3}}{2}, M_{18}=D_{4}\tilde{F}^{2}\tilde{H}_{3}, M_{20}=N^{2}\tilde{F}^{2}\tilde{H}_{3}(D_{5}-1),$$

$$M_{19}=N^{2}\tilde{F}^{2}\tilde{H}_{3}D_{5}, M_{21}=\frac{\tilde{F}^{2}ND_{31}\tilde{H}_{3}}{2}, M_{22}=\frac{\upsilon D_{21}\tilde{H}_{3}^{3}}{2D_{5}}, M_{23}= \frac{\upsilon\tilde{F}D_{4}D_{21}\tilde{H}_{3}^{3}}{D_{5}},$$

$$M_{24}= \frac{\tilde{F}D_{2}N\upsilon D_{21}\tilde{H}_{3}^{3}}{2D_{5}}, M_{25}= \frac{\tilde{F}D_{4}\lambda_{3}\tilde{H}_{3}(\delta+\xi)}{r_{0}D_{2}}, M_{26}=\frac{\tilde{F}D_{5}\delta\lambda_{3}\tilde{H}_{3}}{r_{0}D_{2}}, M_{31}=\frac{\upsilon\tilde{H}_{3}^{3}}{2D_{5}},$$

$$M_{27}=\frac{\tilde{F}^{2}RD_{4}\lambda_{3}\tilde{H}_{3}}{r_{0}D_{2}}, M_{28}=\frac{\tilde{F}^{2}RD_{4}\lambda_{3}\tilde{H}_{3}}{r_{0}D_{2}}, M_{29}=\frac{\tilde{F}^{2}D_{5}NR\lambda_{3}\tilde{H}_{3}}{r_{0}D_{2}}, M_{32}=\frac{x'_{0}\tilde{H}_{3}^{3}}{4D_{5}},$$

$$M_{30}=\frac{\tilde{F}\lambda_{3}\tilde{H}_{3}D_{5}y'_{0}N}{r_{0}D_{2}}, M_{33}=\frac{\tilde{H}_{3}^{3}}{4D_{5}}, M_{34}=\frac{z'_{0}\tilde{H}_{3}}{D_{5}}, M_{35}=\frac{D_{11}\tilde{F}^{2}N^{2}\tilde{H}_{3}\xi_{2}}{D_{3}r_{0}},$$

# Appendix C

$$Q_{1}=\frac{\tilde{F}^{2}N(D_{11}^{2}+D_{31}D_{3})}{2\mu^{2}D_{11}D_{3}}, Q_{2}=\frac{\tilde{F}^{2}N(D_{11}^{2}+D_{3}D_{31})}{6D_{3}D_{11}}, Q_{3}=\tilde{F}\Lambda_{1}\xi_{2}\mu^{-2}(\mu^{2}-1),$$

$$Q_{4}=\Lambda_{1}(\mu^{2}-1)\xi, Q_{5}=\Lambda_{1}(\mu^{2}-1)\xi_{1}\tilde{H}_{3}, Q_{6}=\frac{F\tilde{H}_{3}^{2}D_{1}\upsilon N(1+D_{5}+D_{5}D_{21}-D_{31})}{4\mu},$$

$$Q_{7}=\frac{F\tilde{H}_{3}D_{4}D_{21}\upsilon\lambda_{3}}{4\mu D_{1}r_{0}}, Q_{8}=\frac{F\tilde{H}_{3}x'_{0}N\lambda_{3}}{2\mu D_{1}r_{0}}, Q_{9}=\frac{F\tilde{H}_{3}Ny'_{0}(D_{4}D_{11}+1)}{2D_{4}\mu},$$

$$Q_{10}=\frac{F^{2}\tilde{H}_{3}^{2}D_{4}^{2}\upsilon^{2}\lambda_{3}^{2}D_{11}^{2}}{16\mu D_{2}^{2}r_{0}^{2}}, Q_{10}=\frac{F\tilde{H}_{3}D_{11}x'_{0}N}{2\mu}, Q_{11}=\frac{\upsilon D_{1}D_{4}\tilde{H}_{3}^{2}(D_{21}+1)}{2\mu},$$

$$Q_{12}=\frac{\upsilon ND_{1}^{2}\tilde{H}_{3}^{2}(1+D_{5}+D_{5}D_{21}-D_{31})}{4\mu D_{1}}, Q_{13}=\frac{\delta D_{2}(1+D_{3})}{2\mu D_{1}}, Q_{14}=\frac{D_{4}\lambda_{3}\tilde{H}_{3}x'_{0}}{2D_{5}D_{1}\mu r_{0}},$$

$$Q_{15}=\frac{N\lambda_{3}\tilde{H}_{3}x'_{0}}{2D_{1}\mu r_{0}}, Q_{16}=\frac{D_{4}\upsilon\lambda_{3}D_{21}\tilde{H}_{3}}{4\mu D_{1}r_{0}}, Q_{17}=\frac{\upsilon\lambda_{3}D_{21}\tilde{H}_{3}D_{4}}{4\mu D_{1}r_{0}}, Q_{18}=\frac{\lambda_{3}D_{21}\tilde{H}_{3}x'_{0}}{2\mu D_{1}r_{0}},$$

$$Q_{19}=\frac{D_{4}^{2}\tilde{F}\upsilon^{2}\lambda_{3}^{2}D_{11}^{2}\tilde{H}_{3}^{2}}{32\mu D_{2}^{2}r_{0}^{2}}, Q_{20}=\frac{D_{11}N\tilde{H}_{3}y'_{0}}{2\mu}, Q_{21}=\frac{D_{11}N\tilde{H}_{3}x'_{0}}{2\mu}, Q_{22}=\frac{D_{11}(\upsilon D_{2}+\delta D_{3})}{2\mu D_{2}},$$

$$Q_{23}=\frac{D_{11}^{2}D_{1}D_{4}\upsilon D_{2}\tilde{H}_{3}^{2}r_{0}}{\mu}, Q_{24}=\frac{2D_{1}D_{4}^{2}D_{2}r_{0}x'_{0}\tilde{H}_{3}D_{11}^{2}}{D_{5}\mu}, Q_{25}=\frac{\upsilon D_{2}D_{11}\tilde{H}_{3}D_{4}}{2\mu},$$

$$Q_{26}=\frac{\upsilon D_{2}D_{11}\tilde{H}_{3}D_{5}}{2\mu}, Q_{27}=\frac{D_{2}D_{11}\tilde{H}_{3}x'_{0}}{\mu}, Q_{28}=\frac{D_{2}D_{11}\tilde{H}_{3}y'_{0}}{\mu}, Q_{29}=\frac{\lambda_{3}D_{21}\tilde{H}_{3}y'_{0}}{2\mu D_{1}r_{0}},$$

$$Q_{30}=\frac{D_{11}\lambda_{3}\tilde{H}_{3}x'_{0}}{2\mu D_{2}r_{0}}, Q_{31}=\frac{D_{11}\lambda_{3}\tilde{H}_{3}(D_{5}\upsilon+2y'_{0})}{4\mu D_{2}r_{0}}, Q_{32}=\frac{D_{4}\upsilon\chi\tilde{H}_{3}}{2\mu}, Q_{33}=\frac{D_{5}\upsilon\chi\tilde{H}_{3}^{2}}{2\mu},$$

$$Q_{34}=\frac{\chi\tilde{H}_{3}^{2}x'_{0}}{\mu}, Q_{35}=\frac{Ny'_{0}}{2D_{4}\mu}, Q_{36}=\frac{y'_{0}(1+2D_{4}\chi)}{2D_{4}\mu}, Q_{37}=\frac{\Lambda_{1}\xi_{2}(\mu^{2}-1)}{2\mu},$$

$$Q_{38}=\frac{\upsilon\lambda_{3}D_{21}\tilde{H}_{3}D_{5}}{4\mu D_{1}r_{0}}, Q_{39}=\frac{FD_{4}}{2}, Q_{40}=\frac{FD_{5}N^{2}}{2}, Q_{41}=\upsilon D_{21}D_{4}F\tilde{H}_{3},$$

$$Q_{42}=\frac{\delta D_{1}D_{5}+D_{2}z'_{0}-\upsilon D_{21}D_{2}D_{5}}{2D_{2}D_{5}}, Q_{43}=\frac{\tilde{H}_{3}(D_{4}\upsilon+2x'_{0})+\Lambda_{1}\xi_{1}(\mu^{2}-1)}{2}.$$

# Appendix D

$$S_{1}=\upsilon(1-\frac{D_{1}}{\mu^{2}}-\frac{D_{5}D_{21}\lambda_{3}}{\mu^{2}D_{2}D_{1}r_{0}}-\frac{192\lambda_{3}z'_{0}}{\upsilon D_{2}D_{1}r_{0}}), S_{2}=\frac{\tilde{H}_{3}z'_{0}}{\mu^{2}D_{4}}, S_{3}=\frac{\upsilon D_{1}D_{4}\tilde{F}\tilde{H}_{3}^{2}(D_{21}+1)}{\mu^{2}},$$

$$S_{4}=\frac{\lambda_{3}\tilde{F}^{3}D_{5}D_{21}(D_{4}-D_{5}N^{2})}{64\mu^{2}D_{2}D_{1}r_{0}}, S_{5}=\frac{D_{4}\tilde{F}\tilde{H}_{3}x'_{0}\lambda_{3}}{\mu^{2}D_{2}D_{1}r_{0}}, S_{6}=\frac{D_{11}\tilde{F}^{2}N^{2}y'_{0}}{3\mu^{2}D_{4}D_{3}r_{0}},$$

$$S_{7}=\frac{D_{11}\tilde{F}^{2}N^{2}\tilde{H}_{3}\xi_{2}}{3\mu^{2}D_{3}r_{0}}, S_{8}=\frac{\tilde{F}^{3}\chi(D_{4}-D_{5}N^{2})}{32\mu^{2}}, S_{9}=\frac{\tilde{F}^{3}\chi(D_{4}-D_{5}N^{2})}{8\mu},$$

$$S_{10}=\frac{D_{11}\tilde{F}^{3}\lambda_{3}(D_{4}-D_{5}N^{2})}{64\mu^{2}D_{2}}, S_{11}=\frac{\tilde{F}^{2}D_{2}(D_{4}-D_{5}N^{2})}{32\mu^{2}D_{3}}, S_{12}=\frac{2\tilde{H}_{3}x'_{0}}{\mu^{2}D_{3}},$$

$$S_{13}=\frac{\tilde{H}_{3}x'_{0}\tilde{F}\upsilon\Lambda_{2}}{3\mu^{2}D_{3}}, S_{14}=\frac{\tilde{F}^{2}N^{2}D_{31}}{16\mu^{2}D_{3}}, S_{15}=\frac{\tilde{F}^{2}N^{2}D_{31}}{32\mu^{2}D_{3}}, S_{16}=\frac{\Omega_{1}\tilde{F}^{2}N^{2}D_{31}}{8\mu^{2}D_{3}},$$

$$S_{17}=\frac{\tilde{F}N^{2}D_{31}y'_{0}}{3\mu^{2}D_{4}D_{3}}, S_{18}=\frac{\tilde{F}N^{2}D_{31}\tilde{H}_{3}\xi_{2}}{3\mu^{2}D_{3}}, S_{19}=\Lambda_{1}\xi\left(1-\mu^{2}\right)-\frac{x'_{0}}{D_{5}},$$

$$S_{20}=\frac{\tilde{F}^{2}\tilde{H}_{3}[D_{4}-N^{2}\left(1-D_{5}\right)]}{4}, S_{21}=\frac{\tilde{F}^{2}\tilde{H}_{3}(D_{4}+N^{2}-D_{5}N^{2})}{8}, S_{22}=\frac{\tilde{F}^{2}\tilde{H}_{3}N}{8},$$

$$S_{23}=\frac{\tilde{F}^{2}\tilde{H}_{3}ND_{31}}{16}, S_{24}=\frac{\upsilon\tilde{H}_{3}^{3}}{32}, S_{25}=\frac{\tilde{H}_{3}^{2}D_{4}\upsilon}{6}, S_{26}=\frac{\tilde{H}_{3}^{2}D_{4}D_{21}\tilde{F}\upsilon}{3},$$

$$S_{27}=\frac{\tilde{H}_{3}^{2}D_{21}\tilde{F}\upsilon D_{5}N}{3}, S_{28}=\tilde{H}_{3}^{2}x'_{0} , S_{29}=\frac{\tilde{H}_{3}^{2}(D_{5}\upsilon+3y'_{0})}{6}, S_{30}=\frac{\tilde{F}^{2}\lambda_{3}\tilde{H}_{3}RD_{4}}{8D_{2}r_{0}},$$

$$S_{31}=\frac{\tilde{F}^{2}\lambda_{3}\tilde{H}_{3}D_{4}R}{2D_{2}r_{0}}, S_{32}=\frac{\tilde{F}^{2}\lambda_{3}\tilde{H}_{3}RD_{5}N}{8D_{2}r_{0}}, S_{33}=\frac{\tilde{F}\lambda_{3}\tilde{H}_{3}D_{4}(\delta+\xi)}{2D_{2}r_{0}}, S_{34}=\frac{\tilde{F}\lambda_{3}\tilde{H}_{3}\delta D_{5}}{4D_{2}r_{0}},$$

$$S_{35}=\frac{\tilde{F}\lambda_{3}\tilde{H}_{3}D_{5}Ny'_{0}}{4D_{4}D_{2}r_{0}}, S_{36}=\frac{\lambda_{3}\tilde{F}^{3}(D_{4}\Lambda_{1}+D_{5}N\Lambda_{2})}{2D_{2}r_{0}}, S_{37}=\frac{\tilde{F}\lambda_{3}\tilde{H}_{3}^{2}(D_{4}\xi_{1}+D_{5}N\xi_{2})}{6D_{2}r_{0}},$$

$$S_{38}=\frac{\tilde{F}^{3}(D_{4}-D_{5}N^{2})}{2D_{1}}, S_{39}=\frac{\tilde{F}N\delta\Lambda_{1}D_{2}}{D_{1}}, S_{40}=\frac{\tilde{F}\tilde{H}_{3}D_{4}\upsilon}{2D_{1}}, S_{41}=\frac{\tilde{F}\tilde{H}_{3}D_{5}\upsilon}{2D_{1}},$$

$$S_{42}=\frac{\tilde{F}\tilde{H}_{3}x'_{0}}{D_{1}}, S_{43}=\frac{\tilde{F}\tilde{H}_{3}y'_{0}}{D_{1}}, S_{44}=\frac{\tilde{F}^{2}\delta D_{3}(D_{4}-D_{5}N^{2})}{D_{1}^{2}}, S_{45}=\frac{D_{4}\upsilon\tilde{H}_{3}\delta D_{3}}{D_{1}^{2}},$$

$$S_{46}=\frac{D_{4}\tilde{F}\tilde{H}_{3}\delta D_{3}}{D_{1}^{2}}, S_{47}=\frac{D_{4}\upsilon\tilde{H}_{3}\delta D_{3}}{D_{1}^{2}}, S_{48}=\frac{\tilde{F}D_{4}\tilde{H}_{3}\delta D_{3}}{D_{1}^{2}}, S_{49}=\frac{D_{5}\upsilon\tilde{H}_{3}\delta D_{3}}{D_{1}^{2}},$$

$$S_{50}=\frac{\tilde{F}ND_{5}\tilde{H}_{3}\delta D_{3}}{D_{1}^{2}}, S_{51}=\frac{2x'_{0}\tilde{H}_{3}\delta D_{3}}{D_{1}^{2}}, S_{52}=\frac{2y'_{0}\tilde{H}_{3}\delta D_{3}}{D_{1}^{2}}, S_{53}=\frac{Ny'_{0}}{D_{4}},$$

$$S_{54}=\tilde{F}^{3}N(D_{4}-D_{5}N^{2}), S_{55}=\Lambda_{1}z'_{0}\tilde{F}, S_{56}=\tilde{H}_{3}D_{11}N\upsilon, S_{57}=\tilde{F}\upsilon N\tilde{H}_{3}D_{4},$$

$$S_{58}=\tilde{F}\upsilon N\tilde{H}_{3}D_{5}, S_{59}=2\tilde{F}N\tilde{H}_{3}x'_{0}, S_{60}=\tilde{F}N\tilde{H}_{3}y'_{0}, S_{61}=\tilde{F}N\tilde{H}_{3}\xi_{2},$$

$$S_{62}=\frac{N\tilde{H}_{3}\tilde{F}y'_{0}(2D_{4}+D_{5}N)}{D_{4} }, S_{63}=\frac{\tilde{F}^{2}(D_{5}N^{2}-D_{4})}{2}, S_{64}=\frac{\tilde{H}_{3}(\upsilon D_{4}+2x'_{0})}{2},$$

$$S_{65}=\frac{\tilde{H}_{3}(\upsilon D_{5}+2y'_{0})}{2}, S_{66}=\frac{y'_{0}N}{D_{4}}, S_{67}=\frac{\tilde{H}_{3}^{2}(\upsilon D_{4}+x'_{0})}{2}, S_{68}=\frac{\tilde{F}^{2}D_{5}X^{2}\tilde{H}_{3}}{2},$$

$$S_{69}=\frac{\tilde{H}_{3}^{2}(\upsilon D_{5}+2y'_{0})}{2}, S_{70}=\frac{\tilde{F}^{2}D_{4}\tilde{H}_{3}}{2}, S_{71}=\tilde{H}_{3}\xi_{2}N, S_{72}=\frac{D_{4}\tilde{F}^{2}N\tilde{H}_{3}}{2},$$

$$S_{73}=\tilde{F}\tilde{H}_{3}D_{5}N, S_{74}=\tilde{F}\tilde{H}_{3}D_{4}, S_{75}=K_{22}^{(0)}-\frac{K_{11}^{(0)}}{2},$$

$$S_{76}=\frac{D_{5}[2\tilde{H}_{3}x'_{0}-\Lambda_{1}\xi_{1}(1-\mu^{2})]+z'_{0}}{D_{5}}, S_{77}=\frac{\upsilon(D_{31}\tilde{H}_{3}^{2}-2D_{11})}{2},$$

# Appendix E

$$V_{1}=\frac{\tilde{F}\tilde{H}_{3}r_{0}\gamma_{0}(1+\gamma_{0}^{2}+\gamma_{0}^{4})}{\mu}, V_{2}=\frac{r_{0}\gamma_{0}\tilde{H}_{3}\xi(1+\gamma_{0}^{2}+\gamma_{0}^{4})}{\mu}, V_{3}=\frac{r_{0}\gamma_{0}\tilde{H}_{3}y'_{0}N(1+\gamma_{0}^{2}+\gamma_{0}^{4})}{D_{4}\mu},$$

$$V_{4}=\frac{r_{0}\gamma_{0}\tilde{F}^{2}(\Lambda_{1}-N\Lambda_{2})(1+\gamma_{0}^{2}+\gamma_{0}^{4})}{4\mu}, V_{5}=\frac{r_{0}\gamma_{0}\tilde{H}_{3}^{2}\xi_{2}N}{D_{4}\mu}, V_{6}=\frac{r_{0}\gamma_{0}\tilde{H}_{3}^{2}\xi_{1}}{D_{4}\mu},$$

$$V_{7}=\frac{r_{0}\gamma_{0}^{5}\tilde{F}^{2}(D_{4}+D_{5}N^{2})}{D_{4}\mu}+\frac{r_{0}\gamma_{0}^{3}\tilde{F}^{2}(D_{4}+D_{5}N^{2})}{2D_{4}\mu}, V_{8}=\frac{r_{0}\gamma_{0}^{5}\tilde{F}^{2}(D_{4}-D_{5}N^{2})}{D_{4}\mu},$$

$$V_{9}=\frac{r_{0}\gamma_{0}^{5}\tilde{F}^{2}N(D_{4}+D_{5})}{2D_{4}\mu}, V_{10}=\frac{4r_{0}\gamma_{0}^{5}\tilde{F}^{2}N}{\mu}, V_{11}=\frac{r_{0}\gamma_{0}^{5}\tilde{F}^{2}(D_{4}-D_{5}N^{2})}{2D_{4}\mu},$$

$$V_{12}=\frac{4r_{0}\gamma_{0}^{5}\tilde{F}^{2}}{\mu}, V_{13}=\frac{r_{0}\gamma_{0}^{5}\tilde{F}^{2}(D_{4}+D_{5}N^{2})}{2D_{4}\mu}, V_{14}=\frac{r_{0}\gamma_{0}^{5}\xi_{1}}{2D_{4}\mu}, V_{15}=\frac{2r_{0}\gamma_{0}^{5}N\xi_{2}}{D_{4}\mu},$$

$$V_{16}=\frac{r_{0}\gamma_{0}^{3}\tilde{F}^{2}(D_{4}-D_{5}N^{2})}{2D_{4}\mu}, V_{17}=\frac{r_{0}\gamma_{0}^{3}\tilde{F}^{2}N(D_{4}+D_{5})}{4D_{4}\mu}, V_{18}=\frac{2r_{0}\gamma_{0}^{3}\tilde{F}^{2}N}{\mu},$$

$$V_{19}=\frac{2r_{0}\gamma_{0}^{3}\tilde{F}^{2}}{\mu}, V_{20}=\frac{r_{0}\gamma_{0}^{3}\tilde{F}^{2}(D_{4}-D_{5}N^{2})}{4D_{4}\mu}, V_{21}=\frac{r_{0}\gamma_{0}^{3}\tilde{F}^{2}(D_{4}+D_{5}N^{2})}{4D_{4}\mu},$$

$$V_{22}=\frac{r_{0}\gamma_{0}^{3}\xi_{1}}{2D_{4}\mu}, V_{23}=\frac{r_{0}\gamma_{0}^{3}N\xi_{2}}{D_{4}\mu}, V_{24}=\tilde{F}\tilde{H}_{3}r_{0}\gamma_{0}^{2}(1+\gamma_{0}^{2}+\gamma_{0}^{4}), V_{25}=\frac{\tilde{H}_{3}D_{4}\upsilon D_{5}r_{0}}{2},$$

$$V_{26}=\frac{\tilde{H}_{3}D_{4}\upsilon r_{0}}{2}, V_{27}=\tilde{H}_{3}x'_{0}r_{0}, V_{28}=\tilde{H}_{3}\gamma_{0}^{2}r_{0}\xi(1+\gamma_{0}^{2}+\gamma_{0}^{4}), V_{29}=\tilde{H}_{3}y'_{0}r_{0},$$

$$V_{30}=\frac{\tilde{H}_{3}y'_{0}r_{0}N\gamma_{0}^{2}(1+\gamma_{0}^{2}+\gamma_{0}^{4})}{D_{4}}, V_{31}=\frac{\tilde{F}^{2}r_{0}(D_{4}-D_{5}N^{2})}{2}, V_{32}=\frac{\tilde{F}^{2}D_{4}\tilde{H}_{3}^{2}\gamma_{0}^{2}r_{0}}{8},$$

$$V_{33}=\tilde{F}^{2}r_{0}\gamma_{0}^{2}(1+\gamma_{0}^{2}+\gamma_{0}^{4})(\Lambda_{1}-N\Lambda_{2}), V_{34}=\frac{D_{5}\tilde{F}^{2}X^{2}\tilde{H}_{3}^{2}\gamma_{0}^{2}r_{0}}{8}, V_{35}=\frac{3\tilde{F}^{2}D_{4}\tilde{H}_{3}^{2}\gamma_{0}^{4}r_{0}}{8},$$

$$V_{36}=\frac{3\tilde{F}^{2}D_{4}\tilde{H}_{3}^{2}\gamma_{0}^{4}r_{0}}{8}, V_{37}=\frac{3D_{5}\tilde{F}^{2}N^{2}\tilde{H}_{3}^{2}\gamma_{0}^{4}r_{0}}{8}, V_{38}=\frac{5D_{4}\tilde{F}^{2}\tilde{H}_{3}^{2}\gamma_{0}^{6}r_{0}}{8},$$

$$V_{39}=\frac{5D_{5}\tilde{F}^{2}X^{2}\tilde{H}_{3}^{2}\gamma_{0}^{6}r_{0}}{4}, V_{40}=\frac{5D_{5}\tilde{F}^{2}N^{2}\tilde{H}_{3}^{2}\gamma_{0}^{6}r_{0}}{8}, V_{41}=\frac{\tilde{F}^{2}D_{4}\tilde{H}_{3}^{2}\gamma_{0}^{2}r_{0}(1+3\gamma_{0}^{2}+5\gamma_{0}^{4})}{2},$$

$$V_{42}=\frac{D_{4}\tilde{F}^{2}\tilde{H}_{3}^{2}\gamma_{0}^{2}r_{0}N(1+3\gamma_{0}^{2}+5\gamma_{0}^{4})}{2}, V_{43}=\frac{\tilde{F}^{2}\tilde{H}_{3}^{2}\gamma_{0}^{2}r_{0}(D_{4}-D_{5}N^{2})(1+3\gamma_{0}^{2}+5\gamma_{0}^{4})}{4},$$

$$V_{44}=\frac{\tilde{F}^{2}\tilde{H}_{3}^{2}\gamma_{0}^{2}r_{0}(D_{4}+D_{5}N^{2})(1+3\gamma_{0}^{2}+5\gamma_{0}^{4})}{8}, V_{45}=\frac{N\tilde{F}^{2}D_{4}\tilde{H}_{3}^{2}\gamma_{0}^{2}r_{0}(D_{4}+D_{5})}{8},$$

$$V_{46}=\frac{\tilde{F}^{2}\tilde{H}_{3}^{2}\gamma_{0}^{2}r_{0}N(D_{4}+D_{5})(1+3\gamma_{0}^{2}+5\gamma_{0}^{4})}{8}, V_{47}=\frac{\tilde{H}_{3}^{2}\gamma_{0}^{2}r_{0}\xi_{1}(1-\gamma_{0}^{2}-\gamma_{0}^{4})}{4},$$

$$V_{48}=\frac{\tilde{H}_{3}^{2}\gamma_{0}^{2}r_{0}N\xi_{2}(1+\gamma_{0}^{2}+\gamma_{0}^{4})}{4}.$$
